# A comprehensive p75 neurotrophin receptor gene network and pathway analyses identifying new target genes

**DOI:** 10.1038/s41598-020-72061-z

**Published:** 2020-09-11

**Authors:** Antti Sajanti, Seán B. Lyne, Romuald Girard, Janek Frantzén, Tomi Rantamäki, Iiro Heino, Ying Cao, Cassiano Diniz, Juzoh Umemori, Yan Li, Riikka Takala, Jussi P. Posti, Susanna Roine, Fredrika Koskimäki, Melissa Rahi, Jaakko Rinne, Eero Castrén, Janne Koskimäki

**Affiliations:** 1 Division of Clinical Neurosciences, Department of Neurosurgery, Turku University Hospital and University of Turku, Hämeentie 11, P.O. Box 52, 20521 Turku, Finland; 2 Neurovascular Surgery Program, Section of Neurosurgery, The University of Chicago Medicine and Biological Sciences, 5841 S. Maryland, Chicago, IL 60637 USA; 3 Laboratory of Neurotherapeutics, Molecular and Integrative Biosciences Research Programme, Faculty of Biological and Environmental Sciences and Drug Research Program, Division of Pharmacology and Pharmacotherapy, Faculty of Pharmacy, University of Helsinki, Helsinki, Finland; 4 Neuroscience Center, HiLIFE, University of Helsinki, Box 63, 00014 Helsinki, Finland; 5 Center for Research Informatics, The University of Chicago, Chicago, IL USA; 6 Perioperative Services, Intensive Care and Pain Medicine, Turku University Hospital, POB 52, 20521 Turku, Finland; 7 Department of Anaesthesiology and Intensive Care, University of Turku, Turku, Finland; 8 Division of Clinical Neurosciences, Department of Cerebrovascular Diseases, Turku University Hospital and University of Turku, Hämeentie 11, P.O. Box 52, 20521 Turku, Finland; 9 Department of Psychiatry, Central Hospital of Southern Ostrobothnia, Hanneksenrinne 7, 60220 Seinäjoki, Finland

**Keywords:** Diseases of the nervous system, Molecular neuroscience

## Abstract

P75 neurotrophic receptor (p75NTR) is an important receptor for the role of neurotrophins in modulating brain plasticity and apoptosis. The current understanding of the role of p75NTR in cellular adaptation following pathological insults remains blurred, which makes p75NTR’s related signaling networks an interesting and challenging initial point of investigation. We identified *p75NTR* and related genes through extensive data mining of a PubMed literature search including published works related to p75NTR from the past 20 years. Bioinformatic network and pathway analyses of identified genes (n = 235) were performed using ReactomeFIViz in Cytoscape based on the highly reliable Reactome functional interaction network algorithm. This approach merges interactions extracted from human curated pathways with predicted interactions from machine learning. Genome-wide pathway analysis showed total of 16 enriched hierarchical clusters. A total of 278 enriched single pathways were also identified (p < 0.05, false discovery rate corrected). Gene network analyses showed multiple known and new targets in the *p75NTR* gene network. This study provides a comprehensive analysis and investigation into the current knowledge of p75NTR signaling networks and pathways. These results also identify several genes and their respective protein products as involved in the p75NTR network, which have not previously been clearly studied in this pathway. These results can be used to generate novel hypotheses to gain a greater understanding of p75NTR in acute brain injuries, neurodegenerative diseases and general response to cellular damage.

## Introduction

Neurotrophins are secreted dimeric proteins, including BDNF, the most well-known, as well as nerve growth factor (NGF), neurotrophin-3 (NT3), and neurotrophin-4 (NT4)^1,2^. These neurotrophins and their receptors function in an extensively well-regulated mechanism resulting in a delicate equilibrium that is highly responsive to nervous system injury and change^3^. Neurotrophins bind to two distinct types of receptors including p75 neurotrophin receptor (p75NTR) with a lower affinity, as well as to the tropomyosin receptor kinases (Trk) receptors, which function as tyrosine kinase receptors with high affinity and selectivity^4^. Precursor forms of neurotrophins acts as potent ligands for p75NTR and can induce apoptosis, while lacking affinity to Trk receptors^5^. The Trk receptors activate numerous downstream pathways including the MEK/MAPK pathway, extracellular signal-regulated kinase (Erk), the PI3K-Akt pathway, and the phospholipase C gamma pathway^6^. In contrast the p75NTR does not have intrinsic tyrosine kinase activity, but rather associates with other transmembrane proteins including the Trks, Nogo and myelin associated glycoprotein (MAG) for its downstream effects^5^.

This modulating role of p75NTR have been shown in several animal model studies. Inhibition of p75NTR and its associated Nogo co-receptor exerts a neuroprotective effect in models of middle cerebral artery occlusion^7^. Similarly, a down-regulation of both Nogo and p75NTR promoted improved stroke recovery in another pre-clinical model of middle cerebral artery occlusion^8^. Nogo associates with p75NTR by MAG, oligodendrocyte myelin glycoprotein or Nogo that activates RhoA leading to inhibition of neurite outgrowth^5^. In-line with downregulation exhibiting possible neuroprotective and stimulatory effects, activation of p75NTR by proneurotrophins in culture induces a pro-apoptotic effect on neurons^9^. This is particularly interesting since cleaved neurotrophins promoted recovery through Trks^9^. In another model of central nervous system (CNS) injury, a small-molecule modulator of the p75NTR receptor demonstrated that modulation of this receptor and its associated pathways results in improved outcomes following traumatic brain injuries^10^. All of these studies point to the strong role of neurotrophins and p75NTR in the response to CNS injury.

Viewing the role of neurotrophins in exhibiting neuroprotective effects from a slightly different viewpoint, it has previously been suggested that Alzheimer’s disease (AD) amyloid β peptide also acts as ligand to p75NTR, but not Trk receptors while inducing neuronal apoptosis^11^. This mechanism of p75NTR mediated apoptosis without Trk receptor activation likely plays a significant role in AD pathogenesis^11^. Interestingly, further findings by Yao et al. (2015) demonstrated physiological neuroprotective effects of p75NTR ectodomain against amyloid β peptide toxicity in the brain of AD patients^12^. In addition, AD patients have increased levels of p75NTR and proBDNF in hippocampal tissue samples and a greater proBDNF/BDNF ratio in cerebrospinal fluid that may result in imbalance of death and survival counter-regulation mechanisms^13^. Of note, in AD an increased level of proNGF leads to p75NTR activation and apoptosis in the absence of TrkA^14^. P75NTR has been shown to have similarities in the biology of Amyloid precursor protein (APP) which plays a significant role in AD patophysiology^15^. Also, patients with amyotrophic lateral sclerosis (ALS), another neurodegenerative disease, revealed highly increased p75NTR ectodomain concentrations in urine when compared to healthy controls. In addition, high concentrations of p75NTR ectodomain correlated to progression of ALS disease^16^.

Understanding the role that p75NTR and associated molecules may play in AD also supports the notion that these molecular mechanisms should not be underestimated in the pathophysiological response to acute brain injury. Especially when considering the significant role that plasticity and associated adaptive mechanisms may play in these insults, including chronic traumatic encephalopathy^15,17,18^. With p75NTR being studied in various different diseases and fields suggests that it may play an important central role in cellular response to injury. The body of evidence surrounding p75NTR is diffusely spread out over numerous fields suggesting that a review of these separate bodies of work in one study may identify new connections, pathways, and mechanisms that were previously identified as isolated characteristics.

Herein, we investigate the published literature through machine-learning approaches to study the role of p75NTR and its related gene networks in an attempt to elucidate new factors that may be important to future development of therapeutic targets. We hypothesized that by using already published results of p75NTR signaling, we would be able to investigate *p75NTR* interaction networks and pathways to understand how these genes form networks, connections, and signaling pathways across the broad literature that involves *p75NTR*. By using machine learning educated linkage gene analysis, we aimed to identify new gene and protein candidates that may be involved in a network with *p75NTR*, but have not been widely identified or established as possible targets in the literature regarding acute and chronic response to cellular injury.

## Materials and methods

### Literature search and identification of target genes

In order to identify *p75NTR* and its related genes, we performed an extensive literature search using PubMed on published articles from the past 20 years (1998/11/13–2018/11/13) related to *p75NTR*. The final query term was: (p75[All Fields] AND ("neurons"[MeSH Terms] OR "neurons"[All Fields] OR "neuron"[All Fields])) OR (p75[All Fields] AND ("brain"[MeSH Terms] OR "brain"[All Fields])) OR (p75[All Fields] AND neural[All Fields]) AND ("1998/11/13"[PDAT] : "2018/11/13"[PDAT]). The query resulted in a total of 2041 publications. We used R statistical software with “PubMed.mineR” package to mine all gene names appearing in the 2041 publication abstracts^19^ (Core-team R 2015). Appropriate gene names were used in accordance with HUGO Gene Nomenclature Committee (HGNC) approved symbols (genenames.org). The results of data mining were adjudicated by the authors JK and AS.

### Bioinformatic analyses

**Figure 1:**
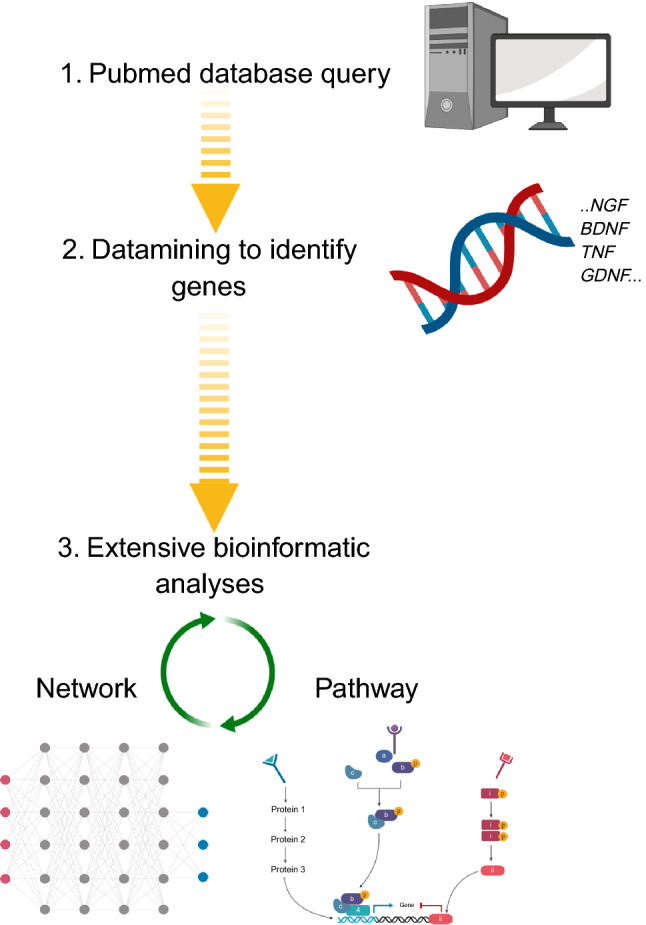
Work-flow diagram. 1 PubMed database queried with inclusive specified search terms. 2 Acquired data mined resulting in target genes. 3 Network analyses performed using a highly reliable algorithm extracted from multiple human-curated pathways. Network analyses followed with pathway enrichment analysis with hypergeometric testing.

A flow of the study is presented in Fig. 1. We used two approaches to study the *p75NTR* gene and its role in gene networks and pathways. Network analyses were performed with ReactomeFIViz in Cytoscape (https://www.cytoscape.org/) based on a highly reliable Reactome functional interaction (FI) network. The entire FI network was constructed by merging interactions extracted from human curated pathways with interactions predicted using a machine learning approach^20,21^. Network analyses were followed with Reactome pathway analyses, and false discovery rate (FDR) corrected p-value < 0.05 was considered to be significant in order to avoid false positive results^22^. Visualization of genome-wide pathway enrichment analysis and generation of hierarchical cluster figure was performed with Reactome analysis tool provided in reactome.org^20^.

### Ethics approval

Institutional ethics board evaluation was not applicable due to the nature of this study. Good scientific practice was used throughout this study.

## Results

### Genes associated to *p75NTR*

With the data mining approach, we identified a total of 235 genes associated to *p75NTR* and its role in neuronal signaling (Tables S1 and S2). Using gene frequencies in the mined literature, the top 20 genes that emerged were *NGF, BDNF*, *TNF*, *MAG*, *GDNF*, *GFAP*, *APP*, *p75NTR*, *TRAF6*, *CNTF*, *EGF*, *EGFR*, *PGP*, *TRPC6*, *PTEN*, *AR*, *CHL1*, *SOX10*, *MBP* and *NTRK1*. A full list of the identified 235 genes is available in the supplemental material.

### Hierarchical genome-wide gene set enrichment analysis

**Figure 2:**
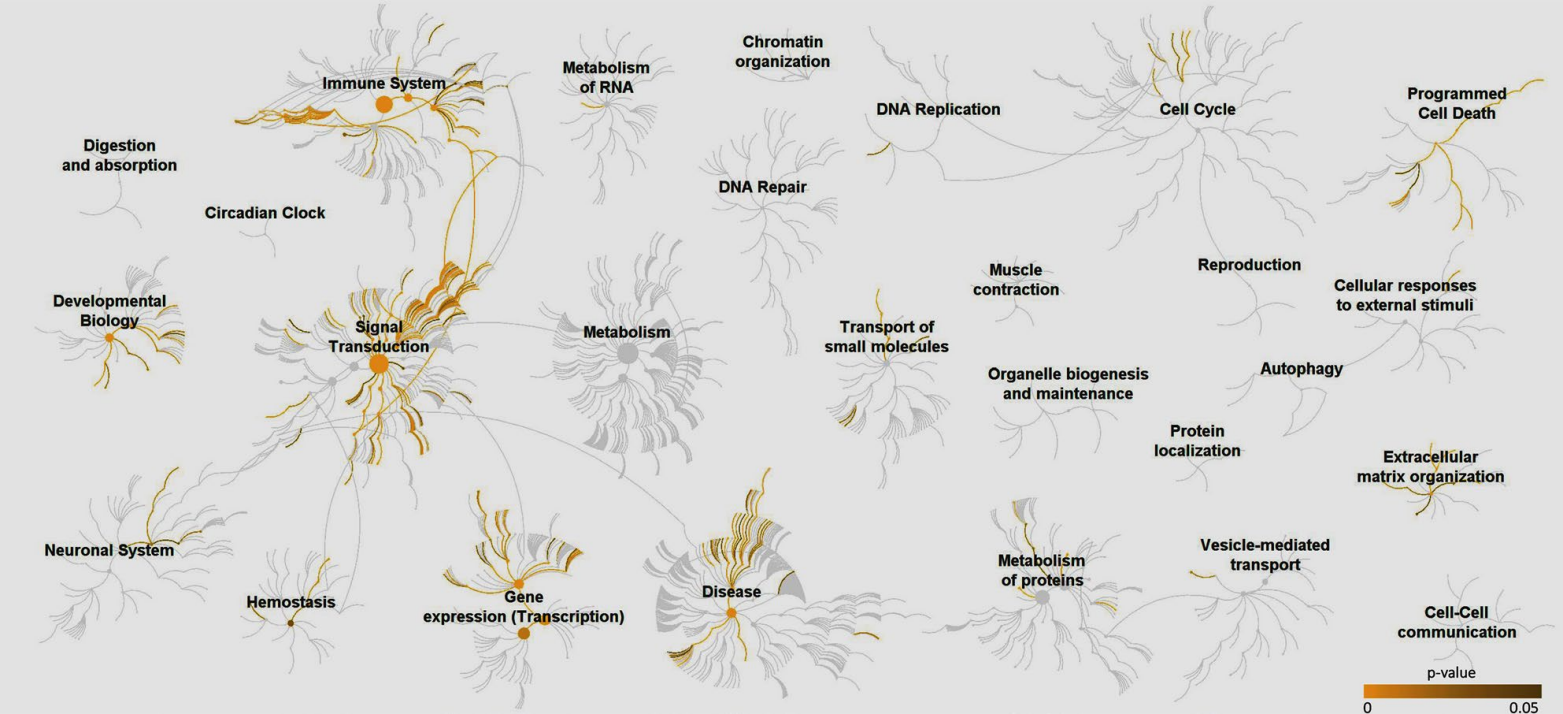
Genome-wide overview of pathway enrichment analysis Enriched pathways are highlighted in yellow. Out of the 27 shown hierarchical clusters, 16 clusters had enriched pathways. Immune system, signal transduction, programmed cell death, developmental biology, gene expression, disease and extracellular matrix organization pathways cluster were the most enriched with numerous pathways.

We used the 235 *p75NTR* related genes to investigate the hierarchical genome-wide pathways and which of these pathways were enriched in known pathways. The enrichment analysis initially demonstrated that *p75NTR* and related genes aggregate in different hierarchical clusters (Fig. 2). Overall, 16 out of 27 hierarchical pathway clusters were enriched with at least one pathway. Hierarchal pathway clusters that included 10 or more enriched pathways were programmed cell death, immune system, signal transduction, developmental biology, gene expression, disease, and extracellular matrix organization.

### Enriched pathways

We further identified enriched pathways related to 235 *p75NTR* related genes without first modeling through hierarchal pathway clusters. Through this analysis, 278 enriched pathways were identified (p < 0.05, FDR corrected, Table S3). The five most enriched pathways (according to FDR corrected p-value) were pathways in cancer, signaling by interleukins, signaling by NGF, melanoma, and proteoglycans in cancer. A list of the top 20 pathways are presented in Table 1. A full list of enriched pathways and individual genes in the nodes are presented in supplemental material.

**Table 1:** Top 20 enriched pathways identified after analyzing 235 *p75NTR* and related genes.

Pathway	Proportion of proteins in pathway	Number of proteins in pathway	Proteins from network	*p*-value, FDR corrected
Pathways in cancer	0.0365	397	38	< 0.0001
Signaling by interleukins	0.0423	460	38	< 0.0001
Signaling by NGF	0.0387	421	36	< 0.0001
Melanoma	0.0065	71	16	< 0.0001
Proteoglycans in cancer	0.0189	205	24	< 0.0001
Signaling by SCF-KIT	0.0267	290	28	< 0.0001
MAPK signaling pathway	0.0235	255	26	< 0.0001
Direct p53 effectors	0.0121	132	19	< 0.0001
Signaling by EGFR	0.0292	317	28	< 0.0001
EGFR tyrosine kinase inhibitor resistance	0.0075	81	15	< 0.0001
Hepatitis B	0.0134	146	19	< 0.0001
p75(NTR)-mediated signaling	0.0063	69	14	< 0.0001
Signaling by PDGF	0.0302	328	27	< 0.0001
Signaling pathways regulating pluripotency of stem cells	0.0131	142	18	< 0.0001
Bladder cancer	0.0038	41	11	< 0.0001
Breast cancer	0.0134	146	18	< 0.0001
PIP3 activates AKT signaling	0.0102	111	16	< 0.0001
Signaling by leptin	0.0192	209	21	< 0.0001
RAF/MAP kinase cascade	0.0185	201	20	< 0.0001

*FDR* false discovery rate.

### Gene network analyses

**Figure 3:**
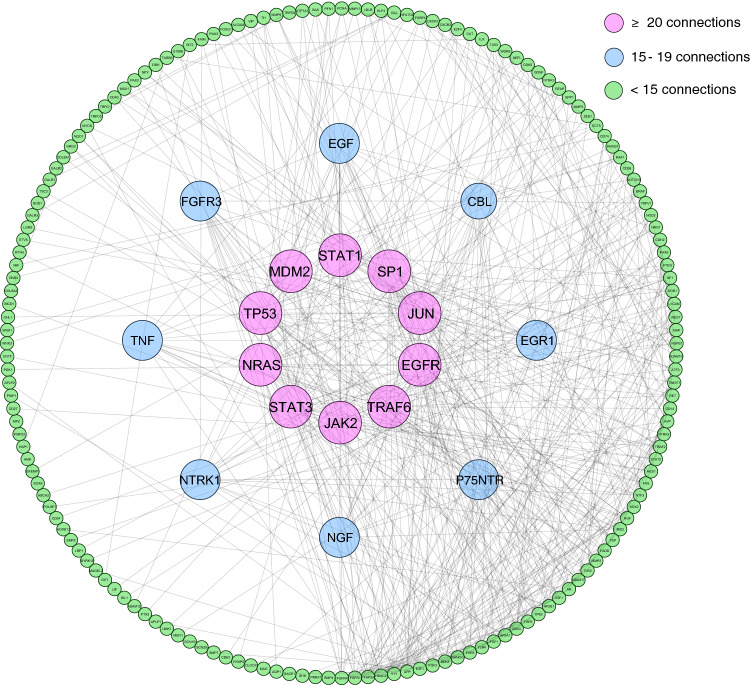
Functional gene interaction network of mined genes related to *p75NTR* A total of 10 genes were highly connected and had 20 or more connections (red). Eight genes showed to have 15 to 19 connections (blue). The remaining genes had less than 15 connections in the network (green).

After pathway analyses, we performed gene network interaction analyses to understand how *p75NTR* and related genes connect to each other with related functions (Fig. 3). Analysis of 235 genes identified 10 highly interconnected genes with more than 20 connections. These highly connected genes were *STAT1, STAT3, SP1, JUN, EGFR, TRAF6, JAK2, NRAS, TP53* and *MDM2*. In addition, *P75NTR, NGF, FGFR3, EGF, TNF, NTRK1, EGR1* and *CBL* were identified to have 15–19 connections. The rest of the mined genes related to *p75NTR* had less than 15 connections in the formed in silico network analysis.

**Figure 4:**
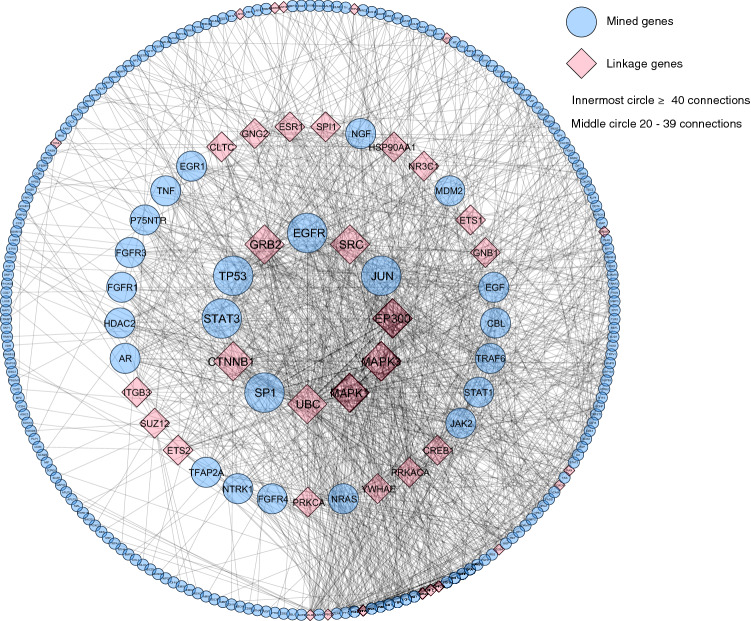
Gene interaction network of mined genes related to *p75NTR* incorporating linkage genes A total of 38 linkage genes were identified, which were functionally related to mined genes. Seven genes identified were highly connected with 40 or more connections. Fifteen genes were identified to have 20–39 connections. Lastly, 16 genes with less than 20 connections were identified. Circles correlate to mined genes, while diamonds correlate to linkage genes.

We performed further gene analyses with the use of linkage genes to identify genes linked to 235 genes through educated machine learning analysis across the Reactome FIViz database. Linkage genes were those genes that were not identified as constituents of the 235 genes. With this approach we identified a total of 38 linkage genes that were mechanistically connected to the mined 235 genes (Fig. 4). Seven of the genes identified were highly connected with 40 or more connections. These genes were *GRB2, SRC, EP300, MAPK1, MAPK3, UBC* and *CTNNB1*. Fifteen linkage genes were identified to have 20–39 connections. The remaining 16 linkage genes had less than 20 connections.

**Figure 5:**
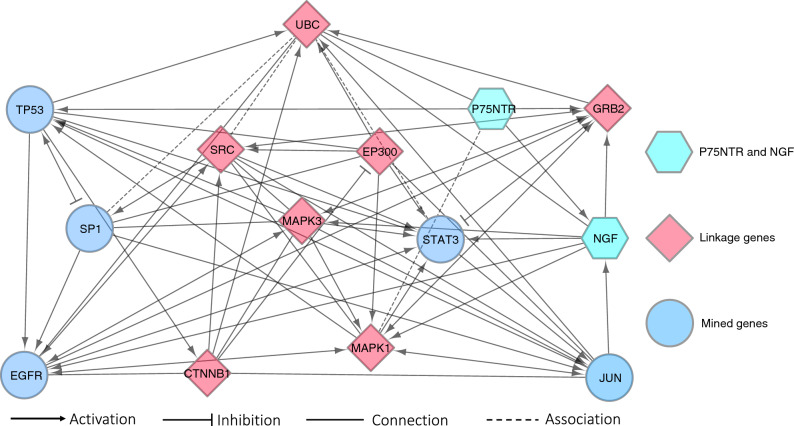
Subnetwork analysis of highly connected genes with linkage genes *P75NTR* and *NGF* were both directly activating *GRB2*. In addition, *p75NTR* had connections to linkage genes *UBC* and *MAPK1*, and, as expected, to its main ligand *NGF* as well as *TP53*. In the same network, several linkage genes *SRC*, *EP300*, *MAPK3* and *CTNNB1* were also identified.

Finally, we performed focused functional interaction subnetwork analyses of identified highly connected (≥ 40) genes and linkage genes in relation to the *p75NTR* and its main ligand *NGF*. *P75NTR* and *NGF* were identified to both activate *GRB2*. In addition, *p75NTR* had direct connection to linkage gene *UBC* and association to *MAPK1*. Furthermore, *p75NTR* had activation connection to its main ligand *NGF* and *TP53*. In the same network, we identified several additional linkage genes including *SRC*, *EP300*, *MAPK3* and *CTNNB1* (Fig. 5).

## Discussion

The complex role of p75NTR in brain plasticity and apoptosis has proven a challenging subject of investigation leaving researchers without a full understanding of the role it plays in various pathophysiological aspects. Herein we investigated previously published literature using data mining and machine learning approaches to gain a better understanding of *p75NTR*’s gene networks and pathways. We identified *p75NTR* related genes, gene networks, and pathways while identifying new associated *p75NTR* network genes. While a portion of these results validate previously published mechanistic links, the advantage of reviewing vast amounts of previously unconnected literature through this approach also identified new genes and proteins for future studies. The pathway analyses provide a general overview of the functions of *p75NTR* in a network with its closely related genes. The data presented here will also serve as a useful resource to the research community in querying potential biomarkers, therapeutic targets, and potential areas of future studies.

Our results obtained from genome-wide cluster and pathway analyses validated the current understanding of p75NTR in that it plays a role in various pathways including programmed cell death, immune system modulation, signal transduction, developmental biology, gene expression regulation, and extracellular matrix organization^2,9,23–27^. The most enriched pathways identified validated the roles of previous studies, such as the finding that p75NTR has been shown to be highly expressed in human cancers including melanoma^26,28,29^. The link of p75NTR to cancer pathology is particularly interesting, given its role in immune modulation, matrix remodeling, and cellular adaptation. However, there are currently no studies demonstrating p75NTR’s effects on modulating the immune response in cancer, nor in acute or chronic brain disease suggesting this area may warrant further investigation, noting also the fact that p75NTR belongs to tumor necrosis factor receptor superfamily. The pathway analyses that resulted provide a general overview of the functions of *p75NTR* in a network with its closely related genes. Overall, 278 enriched pathways and 16 hierarchical pathway clusters were identified generating vast amounts of data that may be useful for validation and future research by other investigators.

The gene network analyses in combination with the focused subnetwork analysis using linkage genes identified functionally related genes that were not part of our original datamined *p75NTR* identified genes. GRB2 has been shown to be directly downstream of TrkA acting as a signaling adaptor protein^30^. Associations or links of *p75NTR* and *GRB2* are not well established. Interestingly, in the brain tissue of scrapie infected rodents, BDNF, TrkB, phospho-TrkB, GRB2 and p75NTR were all significantly down-regulated supporting the direct association identified in our results between *GRB2* and *p75NTR*^31,32^. Viral infection etiology of AD has been a widely known controversial topic. This makes the finding about scrapie viral infection particularly interesting. Results of three independent AD cohorts showed disruption of molecular, genetic, and clinical networks by human herpesvirus^33^. However, the role of p75NTR in possible viral pathophysiology of AD remain unknown, but these results in conjunction with previous works may shed some light generating new hypotheses and studies.

Another linkage gene identified was *UBC,* the gene for polyubiquitin precursor protein*,* which is known to have a role in proteasome degradation^34^. Conjugation of ubiquitins has been well established as highly important for protein degradation and the associated role that plays in larger cellular process such as DNA repair, cell cycle regulation, kinase modification and endocytosis system^34,35^. Loss of *UBC* is associated with the pathophysiological molecular factors of AD via decreased proteasome degradation system, which may be thought of as cells inefficiently removing malfunctioning, damaged, or old proteins^36^. These connections with *p75NTR* underline the important role in AD’s pathophysiology^17^. However, it is important to understand that the role of *p75NTR* in AD pathophysiology is only a model for which *p75NTR* likely acts as a central protein signaler in response to various elements of cellular damage.

MAPK1/ERK2 and MAPK3/ERK1 pathways were also identified and their roles have been reported in apoptosis, neuronal repair, and axonal growth^37,38^. As shown in several studies, MAPKs are downstream targets of Trks and p75NTR^38,39^. Our results suggested that *NGF* and *p75NTR* link with *MAPK1,* and *NGF* activates *MAPK3,* which are in line with previously published studies^39,40^. An interpretation for these interactions in the broader context of a disease can be seen with inhibition of ERK1/2 following the acute phase of stroke promoting long-term functional outcome and enhanced later-stage recovery processes in rats^41^. Other experiments using preclinical rat models have shown how MAPK activity also plays a fundamental role during postnatal neurogenesis when hippocampal apoptosis and synaptogenesis are occurring^42^. The significant connections between MAPK and p75NTR highlight that p75NTR should also be further investigated in relation to these disease processes, as well as finding possible targets for future medical therapy.

In addition, *SRC, CTNNB1,* and *EP300* were identified in the same linkage gene network with *p75NTR*. Interestingly, inhibition of SRC family kinases improved cognitive function in rats after intraventricular hemorrhage^43^. Indeed, these observations further increase the interest of p75NTR and its role following hemorrhagic insult as another form of pathological damage cells suffer acutely. Similarly, *CTNNB1* encodes a β-catenin protein that increases proNGF leading to p75NTR activation ultimately promoting neuronal growth^44^. Further investigation showed that modulation of β-catenin pathway was neuroprotective after intracerebral hemorrhage in rats^45^. The role of p75NTR, however, was not previously studied in these aspects. Our results suggest an association between *CTNNB1* and *p75NTR* possibly demonstrating a substantial role for p75NTR in the stroke response.

Previously, sortilin (*SORT1*), lingo (*LINGO1*) and NRAGE (*MAGED1*) have been linked to p75NTR functions regulating apoptosis, axonal outgrowth and transporting pro-neurotrophins^1,27,39^. The genes did not result in the analyses directly. Interestingly however, *MAGEL2* was identified in our analysis that belong to the same melanoma-associated antigen (MAGE) family than NRAGE, and shares strong homology to NRAGE and other proteins in MAGE-family. As NRAGE is involved in the p75NTR mediated programmed cell death, MAGEL2 is linked to neurodevelopmental disorders such as Prader-Willi syndrome and Schaaf-Yang syndrome. This suggest significant importance of MAGEL2 in neuronal development in humans^46^. Previous animal studies have shown that mTOR and autophagy pathways are dysregulated in *Magel2* null mice models^47^. In our analysis, MAGEL2 was directly linked to transcription factor E2F1 that was directly linked to p75NTR. This suggests a role of necdin-related MAGE proteins in p75NTR functions, supported by preclinical observations^48^.

Two of seven subnetwork linkage genes identified here, namely *UBC*, and *EP300* have not been extensively studied in the context of brain plasticity. These two linkage genes and their encoded proteins could be interesting targets for future studies to explore in patients with acute brain injury or neurodegenerative diseases. This also highlights the benefit of taking large scale approaches through data mining, which allows for an overview of the interactions that may exist between different studies, but have not previously been discovered to exist.

Our study was limited to in silico analyses, and confirmatory in vitro or in vivo analyses were not performed. However, the research approach used herein has the advantage of interrogating a large dataset through a systematical approach which incorporates all currently available knowledge. Analyzing such a search with powerful bioinformatic tools educated with machine learning algorithm from human curated pathways allows for broad investigation of connections that may have previously been missed, or not looked for in the first place. Confirmation of discussed associations are warranted.

## Conclusion

We provide the single largest comprehensive gene and functional network library of *p75NTR*. This study incorporates current knowledge using a large dataset approach that increases the overall understanding of complex *p75NTR* networks. These results suggest both new possible target genes for further investigation in p75NTR research, while also validating previously conducted research in identifying pathways, genes, and clusters that highlight p75NTR’s biological function. These results can be used to generate novel hypotheses to gain a greater understanding of p75NTR in acute or chronic brain injuries, other neurodegenerative diseases, and the general response to cellular injury.

## Supplementary information

Supplementary Information 1.

Supplementary Information 2.

## Data Availability

All data generated or analyzed during this study are included in this manuscript and its supplementary information files. Source data is included as supplementary file and also can be retrieved from PubMed.gov with term: (p75[All Fields] AND ("neurons"[MeSH Terms] OR "neurons"[All Fields] OR "neuron"[All Fields])) OR (p75[All Fields] AND ("brain"[MeSH Terms] OR "brain"[All Fields])) OR (p75[All Fields] AND neural[All Fields]) AND ("1998/11/13"[PDAT]: "2018/11/13"[PDAT]).

## Code availability

Open source R statistical software were used with “PubMed.mineR” package^19^. Figure 1 was generated by using biorender tool (biorender.com). Network and pathway analyses were performed with ReactomeFIViz in Cytoscape software platform (https://www.cytoscape.org/), and respective network figures were generated by the same program tool^20,21^.
