# Supplementary material for: A comprehensive p75 neurotrophin receptor gene network and pathway analyses identifying new target genes
Source: Sci Rep. 2020 Sep 11;10:14984. doi: 10.1038/s41598-020-72061-z (PMC7486379; doi:10.1038/s41598-020-72061-z)
Supplement: Supplementary file 2 — Supplementary Information 2. [file 41598_2020_72061_MOESM2_ESM.docx]

**Supplemental Material**

**A comprehensive p75 neurotrophin receptor gene network and pathway analyses identifying new target genes**

Antti Sajanti^1^, Seán B. Lyne^2^, Romuald Girard^2^, Janek Frantzén^1^, Tomi Rantamäki^3^, Iiro Heino^1^, Ying Cao^2^, Cassiano Diniz^4^, Juzoh Umemori^4^, Yan Li^2,5^, Riikka Takala^6^, Jussi P. Posti^1^, Susanna Roine^7^, Fredrika Koskimäki^7^, Melissa Rahi^1^, Jaakko Rinne^1^, Eero Castrén^4^, Janne Koskimäki^1,8^

^1^Division of Clinical Neurosciences, Department of Neurosurgery, Turku University Hospital and University of Turku, P.O. Box 52, Hämeentie 11, FI-20521, Turku, Finland.

^2^Neurovascular Surgery Program, Section of Neurosurgery, The University of Chicago Medicine and Biological Sciences, 5841 S. Maryland, Chicago, IL 60637, US.

^3^Laboratory of Neurotherapeutics, Molecular and Integrative Biosciences Research Programme, Faculty of Biological and Environmental Sciences and Drug Research Program, Division of Pharmacology and Pharmacotherapy, Faculty of Pharmacy, University of Helsinki, Finland

^4^Neuroscience Center, HiLIFE, University of Helsinki, Box 63, 00014 Helsinki, Finland.

^5^Center for Research Informatics, The University of Chicago, Chicago, IL

^6^Perioperative Services, Intensive Care and Pain Medicine, Turku University Hospital, POB 52, 20521, Turku, Finland and Department of Anaesthesiology and Intensive Care, University of Turku, Turku, Finland.

^7^Division of Clinical Neurosciences, Department of Cerebrovascular Diseases, Turku University Hospital and University of Turku, P.O. Box 52, Hämeentie 11, FI-20521, Turku, Finland.

^8^Department of Psychiatry, Central Hospital of Southern Ostrobothnia, Hanneksenrinne 7, FI-60220, Seinäjoki, Finland.

**Corresponding author:**

Janne Koskimäki, MD, PhD

Division of Clinical Neurosciences, Department of Neurosurgery, Turku University Hospital and University of Turku, P.O. Box 52 (Hämeentie 11), FI-20521, Turku, Finland.

E-mail: jj.koskimaki@gmail.com

Tel: +358 44 213 4939

**Supplemental tables**

| **Table S1. Datamined genes (n=235) and frequencies in the literature. HGNC = HUGO gene nomenclature committee.** | | |
| --- | --- | --- |
| **HGNC gene symbol** | **Gene name** | **Frequency** |
| *NGF* | nerve growth factor (beta polypeptide) | 1702 |
| *BDNF* | brain-derived neurotrophic factor | 1264 |
| *TNF* | tumor necrosis factor | 104 |
| *MAG* | myelin associated glycoprotein | 73 |
| *GDNF* | glial cell derived neurotrophic factor | 60 |
| *GFAP* | glial fibrillary acidic protein | 59 |
| *APP* | amyloid beta (A4) precursor protein | 56 |
| *NGFR* | nerve growth factor receptor | 28 |
| *TRAF6* | TNF receptor-associated factor 6, E3 ubiquitin protein ligase | 26 |
| *CNTF* | ciliary neurotrophic factor | 24 |
| *EGF* | epidermal growth factor | 24 |
| *EGFR* | epidermal growth factor receptor | 23 |
| *PGP* | phosphoglycolate phosphatase | 23 |
| *TRPC6* | transient receptor potential cation channel, subfamily C, member 6 | 18 |
| *PTEN* | phosphatase and tensin homolog | 17 |
| *AR* | androgen receptor | 16 |
| *CHL1* | cell adhesion molecule with homology to L1CAM | 16 |
| *SOX10* | SRY (sex determining region Y)-box 10 | 13 |
| *MBP* | myelin basic protein | 12 |
| *NTRK1* | neurotrophic tyrosine kinase, receptor, type 1 | 12 |
| *NTRK2* | neurotrophic tyrosine kinase, receptor, type 2 | 12 |
| *SOD1* | superoxide dismutase 1, soluble | 12 |
| *AQP4* | aquaporin 4 | 11 |
| *CD44* | CD44 molecule (Indian blood group) | 11 |
| *GAL* | galanin/GMAP prepropeptide | 11 |
| *MIF* | macrophage migration inhibitory factor | 11 |
| *VGF* | VGF nerve growth factor inducible | 11 |
| *AMIGO3* | adhesion molecule with Ig-like domain 3 | 10 |
| *NPY* | neuropeptide Y | 10 |
| *CD34* | CD34 molecule | 9 |
| *GHRH* | growth hormone releasing hormone | 9 |
| *HGF* | hepatocyte growth factor (hepapoietin A; scatter factor) | 9 |
| *LRP1* | low density lipoprotein receptor-related protein 1 | 9 |
| *TRPC1* | transient receptor potential cation channel, subfamily C, member 1 | 9 |
| *VIP* | vasoactive intestinal peptide | 9 |
| *CRABP1* | cellular retinoic acid binding protein 1 | 8 |
| *LDLR* | low density lipoprotein receptor | 8 |
| *LIF* | leukemia inhibitory factor | 8 |
| *PCNA* | proliferating cell nuclear antigen | 8 |
| *TH* | tyrosine hydroxylase | 8 |
| *TLR2* | toll-like receptor 2 | 8 |
| *WNK3* | WNK lysine deficient protein kinase 3 | 8 |
| *FAIM* | Fas apoptotic inhibitory molecule | 7 |
| *FGFR1* | fibroblast growth factor receptor 1 | 7 |
| *HAP1* | huntingtin-associated protein 1 | 7 |
| *HPSE* | heparanase | 7 |
| *NRG1* | Neuregulin 1 | 7 |
| *PMP22* | peripheral myelin protein 22 | 7 |
| *SOX9* | SRY (sex determining region Y)-box 9 | 7 |
| *ABCA2* | ATP-binding cassette, sub-family A (ABC1), member 2 | 6 |
| *ADAM17* | ADAM metallopeptidase domain 17 | 6 |
| *BACE1* | beta-site APP-cleaving enzyme 1 | 6 |
| *CHAT* | choline O-acetyltransferase | 6 |
| *E2F1* | E2F transcription factor 1 | 6 |
| *STAT3* | signal transducer and activator of transcription 3 | 6 |
| *ASIC3* | acid-sensing (proton-gated) ion channel 3 | 5 |
| *BAX* | BCL2-associated X protein | 5 |
| *CBL* | Cbl proto-oncogene, E3 ubiquitin protein ligase | 5 |
| *FGF2* | fibroblast growth factor 2 (basic) | 5 |
| *MAGEL2* | MAGE-like 2 | 5 |
| *MAP1B* | microtubule-associated protein 1B | 5 |
| *MYCN* | v-myc myelocytomatosis viral related oncogene, neuroblastoma derived | 5 |
| *SOX2* | SRY (sex determining region Y)-box 2 | 5 |
| *TRPC5* | transient receptor potential cation channel, subfamily C, member 5 | 5 |
| *ALCAM* | activated leukocyte cell adhesion molecule | 4 |
| *BMP4* | bone morphogenetic protein 4 | 4 |
| *BMP7* | bone morphogenetic protein 7 | 4 |
| *CD36* | CD36 molecule (thrombospondin receptor) | 4 |
| *CD68* | CD68 molecule | 4 |
| *E2F4* | E2F transcription factor 4, p107/p130-binding | 4 |
| *EMX1* | empty spiracles homeobox 1 | 4 |
| *EMX2* | empty spiracles homeobox 2 | 4 |
| *FAS* | Fas (TNF receptor superfamily, member 6) | 4 |
| *FGF8* | fibroblast growth factor 8 (androgen-induced) | 4 |
| *GIT1* | G protein-coupled receptor kinase interacting ArfGAP 1 | 4 |
| *GIT2* | G protein-coupled receptor kinase interacting ArfGAP 2 | 4 |
| *MAL* | mal, T-cell differentiation protein | 4 |
| *NANOG* | Nanog homeobox | 4 |
| *NOTCH3* | notch 3 | 4 |
| *NTRK3* | neurotrophic tyrosine kinase, receptor, type 3 | 4 |
| *PAX6* | paired box 6 | 4 |
| *PDLIM1* | PDZ and LIM domain 1 | 4 |
| *RET* | ret proto-oncogene | 4 |
| *VDR* | vitamin D (1,25- dihydroxyvitamin D3) receptor | 4 |
| *WNK1* | WNK lysine deficient protein kinase 1 | 4 |
| *AQP1* | aquaporin 1 (Colton blood group) | 3 |
| *BICD1* | bicaudal D homolog 1 (Drosophila) | 3 |
| *CLOCK* | clock circadian regulator | 3 |
| *DISC1* | disrupted in schizophrenia 1 | 3 |
| *DMP1* | dentin matrix acidic phosphoprotein 1 | 3 |
| *DYRK1B* | dual-specificity tyrosine-(Y)-phosphorylation regulated kinase 1B | 3 |
| *JAK2* | Janus kinase 2 | 3 |
| *KLF4* | Kruppel-like factor 4 (gut) | 3 |
| *L1CAM* | L1 cell adhesion molecule | 3 |
| *LIMK1* | LIM domain kinase 1 | 3 |
| *MAP2* | microtubule-associated protein 2 | 3 |
| *PAX3* | paired box 3 | 3 |
| *PLP1* | proteolipid protein 1 | 3 |
| *REST* | RE1-silencing transcription factor | 3 |
| *S100B* | S100 calcium binding protein B | 3 |
| *STAT1* | signal transducer and activator of transcription 1, 91kDa | 3 |
| *TRAF2* | TNF receptor-associated factor 2 | 3 |
| *TRAF4* | TNF receptor-associated factor 4 | 3 |
| *AMH* | anti-Mullerian hormone | 2 |
| *AVP* | arginine vasopressin | 2 |
| *BMP2* | bone morphogenetic protein 2 | 2 |
| *CCT5* | chaperonin containing TCP1, subunit 5 (epsilon) | 2 |
| *CD24* | CD24 molecule | 2 |
| *CD47* | CD47 molecule | 2 |
| *CD74* | CD74 molecule, major histocompatibility complex | 2 |
| *CDH2* | cadherin 2, type 1, N-cadherin (neuronal) | 2 |
| *DCX* | doublecortin | 2 |
| *FAAH* | fatty acid amide hydrolase | 2 |
| *FADD* | Fas (TNFRSF6)-associated via death domain | 2 |
| *FGF9* | fibroblast growth factor 9 (glia-activating factor) | 2 |
| *FGFR4* | fibroblast growth factor receptor 4 | 2 |
| *FKBP4* | FK506 binding protein 4, 59kDa | 2 |
| *FNDC5* | fibronectin type III domain containing 5 | 2 |
| *IGF1* | insulin-like growth factor 1 (somatomedin C) | 2 |
| *ILK* | integrin-linked kinase | 2 |
| *MMP2* | matrix metallopeptidase 2 | 2 |
| *MMP9* | matrix metallopeptidase 9 | 2 |
| *NEFL* | neurofilament, light polypeptide | 2 |
| *NF2* | neurofibromin 2 (merlin) | 2 |
| *NQO1* | NAD(P)H dehydrogenase, quinone 1 | 2 |
| *NTF3* | neurotrophin 3 | 2 |
| *PDK1* | pyruvate dehydrogenase kinase, isozyme 1 | 2 |
| *PFN1* | profilin 1 | 2 |
| *PHOX2B* | paired-like homeobox 2b | 2 |
| *SCN8A* | sodium channel, voltage gated, type VIII, alpha subunit | 2 |
| *SCRG1* | stimulator of chondrogenesis 1 | 2 |
| *SORCS2* | sortilin-related VPS10 domain containing receptor 2 | 2 |
| *SP1* | Sp1 transcription factor | 2 |
| *SPTLC1* | serine palmitoyltransferase, long chain base subunit 1 | 2 |
| *SSTR3* | somatostatin receptor 3 | 2 |
| *TBP* | TATA box binding protein | 2 |
| *TFAP2A* | transcription factor AP-2 alpha | 2 |
| *TLR4* | toll-like receptor 4 | 2 |
| *TP63* | tumor protein p63 | 2 |
| *TRPV1* | transient receptor potential cation channel, subfamily V, member 1 | 2 |
| *XIAP* | X-linked inhibitor of apoptosis | 2 |
| *ABCG2* | ATP-binding cassette, sub-family G (WHITE), member 2 | 1 |
| *ADAM10* | ADAM metallopeptidase domain 10 | 1 |
| *ADAM12* | ADAM metallopeptidase domain 12 | 1 |
| *ALDH1L1* | aldehyde dehydrogenase 1 family, member L1 | 1 |
| *ALDH5A1* | aldehyde dehydrogenase 5 family, member A1 | 1 |
| *AP2A1* | adaptor-related protein complex 2, alpha 1 subunit | 1 |
| *AP2B1* | adaptor-related protein complex 2, beta 1 subunit | 1 |
| *APLP1* | amyloid beta (A4) precursor-like protein 1 | 1 |
| *APLP2* | amyloid beta (A4) precursor-like protein 2 | 1 |
| *ATF3* | activating transcription factor 3 | 1 |
| *ATP1A1* | ATPase, Na+/K+ transporting, alpha 1 polypeptide | 1 |
| *BRAF* | v-raf murine sarcoma viral oncogene homolog B1 | 1 |
| *CAT* | catalase | 1 |
| *CCK* | cholecystokinin | 1 |
| *CD14* | CD14 molecule | 1 |
| *CD27* | CD27 molecule | 1 |
| *CD40* | CD40 molecule, TNF receptor superfamily member 5 | 1 |
| *CD63* | CD63 molecule | 1 |
| *CDH19* | cadherin 19, type 2 | 1 |
| *CDK5* | cyclin-dependent kinase 5 | 1 |
| *CNTNAP2* | contactin associated protein-like 2 | 1 |
| *COL8A1* | collagen, type VIII, alpha 1 | 1 |
| *COL8A2* | collagen, type VIII, alpha 2 | 1 |
| *CXCR1* | chemokine (C-X-C motif) receptor 1 | 1 |
| *CXCR2* | chemokine (C-X-C motif) receptor 2 | 1 |
| *DBH* | dopamine beta-hydroxylase (dopamine beta-monooxygenase) | 1 |
| *DHH* | desert hedgehog | 1 |
| *DLX1* | distal-less homeobox 1 | 1 |
| *EFEMP1* | EGF containing fibulin-like extracellular matrix protein 1 | 1 |
| *EGR1* | early growth response 1 | 1 |
| *ETV5* | ets variant 5 | 1 |
| *FASN* | fatty acid synthase | 1 |
| *FGF1* | fibroblast growth factor 1 (acidic) | 1 |
| *FGFR3* | fibroblast growth factor receptor 3 | 1 |
| *FKBP5* | FK506 binding protein 5 | 1 |
| *FOXC2* | forkhead box C2 (MFH-1, mesenchyme forkhead 1) | 1 |
| *FOXG1* | forkhead box G1 | 1 |
| *GALR1* | galanin receptor 1 | 1 |
| *GALR2* | galanin receptor 2 | 1 |
| *GALR3* | galanin receptor 3 | 1 |
| *GAP43* | growth associated protein 43 | 1 |
| *GNB4* | guanine nucleotide binding protein (G protein), beta polypeptide 4 | 1 |
| *HDAC2* | histone deacetylase 2 | 1 |
| *HES1* | hairy and enhancer of split 1, (Drosophila) | 1 |
| *HMGB1* | high mobility group box 1 | 1 |
| *HOXB13* | homeobox B13 | 1 |
| *HOXC4* | homeobox C4 | 1 |
| *HOXD8* | homeobox D8 | 1 |
| *HSPB1* | heat shock 27kDa protein 1 | 1 |
| *IRS2* | insulin receptor substrate 2 | 1 |
| *ISL1* | ISL LIM homeobox 1 | 1 |
| *JUN* | jun proto-oncogene | 1 |
| *LGR5* | leucine-rich repeat containing G protein-coupled receptor 5 | 1 |
| *LHX8* | LIM homeobox 8 | 1 |
| *MDM2* | MDM2 oncogene, E3 ubiquitin protein ligase | 1 |
| *MEN1* | multiple endocrine neoplasia I | 1 |
| *MMP3* | matrix metallopeptidase 3 (stromelysin 1, progelatinase) | 1 |
| *MMP13* | matrix metallopeptidase 13 (collagenase 3) | 1 |
| *MPZ* | myelin protein zero | 1 |
| *MSX1* | msh homeobox 1 | 1 |
| *NES* | nestin | 1 |
| *NF1* | neurofibromin 1 | 1 |
| *NFATC4* | nuclear factor of activated T-cells, cytoplasmic, calcineurin-dependent 4 | 1 |
| *NOD1* | nucleotide-binding oligomerization domain containing 1 | 1 |
| *NOD2* | nucleotide-binding oligomerization domain containing 2 | 1 |
| *NRAS* | neuroblastoma RAS viral (v-ras) oncogene homolog | 1 |
| *NRG3* | neuregulin 3 | 1 |
| *NSF* | N-ethylmaleimide-sensitive factor | 1 |
| *PAX2* | paired box 2 | 1 |
| *PDE4D* | phosphodiesterase 4D, cAMP-specific | 1 |
| *POU3F1* | POU class 3 homeobox 1 | 1 |
| *PRRX1* | paired related homeobox 1 | 1 |
| *PSIP1* | PC4 and SFRS1 interacting protein 1 | 1 |
| *PTX3* | pentraxin 3, long | 1 |
| *RAF1* | v-raf-1 murine leukemia viral oncogene homolog 1 | 1 |
| *RTN4* | reticulon 4 | 1 |
| *SCAP* | SREBF chaperone | 1 |
| *SCN1B* | sodium channel, voltage-gated, type I, beta subunit | 1 |
| *SCN2B* | sodium channel, voltage-gated, type II, beta subunit | 1 |
| *SCN3B* | sodium channel, voltage-gated, type III, beta subunit | 1 |
| *SHH* | sonic hedgehog | 1 |
| *SPP1* | secreted phosphoprotein 1 | 1 |
| *STAT2* | signal transducer and activator of transcription 2, 113kDa | 1 |
| *TCOF1* | Treacher Collins-Franceschetti syndrome 1 | 1 |
| *TLR3* | toll-like receptor 3 | 1 |
| *TNPO3* | transportin 3 | 1 |
| *TP53* | tumor protein p53 | 1 |
| *TRPA1* | transient receptor potential cation channel, subfamily A, member 1 | 1 |
| *TSC2* | tuberous sclerosis 2 | 1 |
| *TUBB3* | tubulin, beta 3 class III | 1 |
| *TWIST1* | twist basic helix-loop-helix transcription factor 1 | 1 |
| *WDR5* | WD repeat domain 5 | 1 |
| *YY1* | YY1 transcription factor | 1 |
| *ZEB1* | zinc finger E-box binding homeobox 1 | 1 |

| **Table S2. Excluded genes (n=43) following review of mined genes** | | | | |
| --- | --- | --- | --- | --- |
| **HGNC gene symbol** | **Gene name** | **Freq.** | **Explanation** |  |
| *CA1* | carbonic anhydrase I | 45 | Cornu ammonis |  |
| *T* | T, brachyury homolog (mouse) | 39 | Author first name abreviated |  |
| *C6* | complement component 6 | 20 | Reference coding |  |
| *MB* | myoglobin | 20 | Reference coding |  |
| *CA3* | carbonic anhydrase III, muscle specific | 19 | Cornu ammonis |  |
| *PC* | pyruvate carboxylase | 16 | Reference coding |  |
| *MPG* | N-methylpurine-DNA glycosylase | 14 | Major pelvic ganglia |  |
| *CP* | ceruloplasmin (ferroxidase) | 11 | Reference coding |  |
| *GCA* | grancalcin, EF-hand calcium binding protein | 11 | Giant cell arteritis |  |
| *TG* | thyroglobulin | 10 | Dynamitin |  |
| *CGN* | cingulin | 9 | Cerebellar granule neurons |  |
| *IVD* | isovaleryl-CoA dehydrogenase | 8 | Intervertebral disc |  |
| *SI* | sucrase-isomaltase (alpha-glucosidase) | 8 | Reference coding |  |
| *SON* | SON DNA binding protein | 8 | Supraoptcic nucleus |  |
| *CA4* | carbonic anhydrase IV | 7 | Cornu ammonis |  |
| *NPS* | neuropeptide S | 6 | Nanoparticles |  |
| *PSD* | pleckstrin and Sec7 domain containing | 6 | Postsynaptic density |  |
| *DSP* | desmoplakin | 5 | Distal sensory polyneuropathy |  |
| *KL* | Klotho | 5 | Kit ligand, and author first name abreviated |  |
| *NCL* | nucleolin | 4 | Neural crest like |  |
| *TEC* | tec protein tyrosine kinase | 4 | Thymic epithelial cells |  |
| *TES* | testis derived transcript (3 LIM domains) | 4 | Tissue engineered skin |  |
| *CA2* | carbonic anhydrase II | 3 | Cornu ammonis |  |
| *ECD* | ecdysoneless homolog (Drosophila) | 3 | Extracellular domain |  |
| *MNT* | MNT, MAX dimerization protein | 3 | Mental nerve transection |  |
| *MSN* | moesin | 3 | Medial septal nucleus |  |
| *GC* | group-specific component (vitamin D binding protein) | 2 | Glugocorticoids, Gustatory cortex |  |
| *MAX* | MYC associated factor X | 2 | Maximum |  |
| *MSC* | musculin | 2 | bone marrow-derived stromal cells |  |
| *PI3* | peptidase inhibitor 3, skin-derived | 2 | PI3-kinase |  |
| *SCD* | stearoyl-CoA desaturase (delta-9-desaturase) | 2 | Sudden cardiac death |  |
| *TNC* | tenascin C | 2 | Trigeminal complex |  |
| *C5* | complement component 5 | 1 | part of author's address |  |
| *C7* | complement component 7 | 1 | C7 nerve root |  |
| *DCT* | dopachrome tautomerase | 1 | Dorsal colum transection |  |
| *F11* | coagulation factor XI | 1 | F11 neuronal hybrid cells |  |
| *FGA* | fibrinogen alpha chain | 1 | First generation antipsychotics |  |
| *IGL* | immunoglobulin lambda locus | 1 | Internal granule layer |  |
| *LTF* | lactotransferrin | 1 | Long term facilitiation |  |
| *MN1* | meningioma (disrupted in balanced translocation) 1 | 1 | Motorneuron 1 cell line |  |
| *PML* | promyelocytic leukemia | 1 | Progresive multifocal leukoencephalopathy |  |
| *RPE* | ribulose-5-phosphate-3-epimerase | 1 | Retinal pigment epithelium |  |
| *SPR* | sepiapterin reductase (7,8-dihydrobiopterin:NADP+ oxidoreductase) | 1 | Surface plasmoid sensor |  |

| **Table S3. Pathway analysis of datamined genes identified 278 statistically enriched pathways** (p < 0.05, false discovery rate (FDR) corrected. (C)=CellMap, (R)=Reactome, (K)=KEGG, (N)=NCI PID, (P)=Panther, and (B=)BioCarta) | | | | | | |  |
| --- | --- | --- | --- | --- | --- | --- | --- |
| **Pathway** | **Ratio of protein in pathway** | **Number of protein in pathway** | **Protein from network** | ***p*-value** | ***p*-value, FDR corrected** | **Nodes** | |
| Pathways in cancer(K) | 0,0365 | 397 | 38 | 2,22E-16 | 1,61E-13 | AR,TP53,FADD,JUN,BRAF,IGF1,RET,SHH,XIAP,EGFR,NRAS,STAT1,STAT3,FAS,FGF1,FGF2,FGF8,FGF9,HGF,RAF1,BMP4,BMP2,GNB4,MDM2,FGFR3,FGFR1,PTEN,MMP2,TRAF2,MMP9,TRAF4,TRAF6,HDAC2,CBL,E2F1,NTRK1,EGF,BAX | |
| Signaling by Interleukins(R) | 0,0423 | 460 | 38 | 2,79E-14 | 1,01E-11 | CNTF,SOX2,TP53,TWIST1,LIF,BRAF,NANOG,RET,NEFL,CD36,JAK2,ZEB1,EGFR,NRAS,STAT1,STAT3,IRS2,FGF1,FGF2,TNF,FGF8,FGF9,HGF,RAF1,GDNF,NF1,FGFR4,FGFR3,FGFR1,MMP2,MMP3,NRG1,MMP9,TRAF6,NOD1,NOD2,CBL,EGF | |
| Signalling by NGF(R) | 0,0387 | 421 | 36 | 5,35E-14 | 1,30E-11 | TP53,NGFR,BRAF,RET,AP2A1,NEFL,JAK2,EGFR,NRAS,STAT3,AP2B1,IRS2,FGF1,FGF2,FGF8,FGF9,HGF,TSC2,MAG,ADAM17,RAF1,RTN4,GDNF,NF1,MDM2,FGFR4,FGFR3,FGFR1,PTEN,NRG1,NGF,TRAF6,HDAC2,NTRK1,NTRK2,EGF | |
| Melanoma(K) | 0,0065 | 71 | 16 | 1,02E-12 | 1,85E-10 | TP53,BRAF,IGF1,EGFR,NRAS,FGF1,FGF2,FGF8,FGF9,HGF,RAF1,MDM2,FGFR1,PTEN,E2F1,EGF | |
| Proteoglycans in cancer(K) | 0,0189 | 205 | 24 | 2,27E-12 | 3,29E-10 | TP53,TWIST1,BRAF,IGF1,NANOG,SHH,TLR4,CD44,TLR2,CD63,EGFR,NRAS,STAT3,FAS,FGF2,TNF,HGF,RAF1,MDM2,HPSE,FGFR1,MMP2,MMP9,CBL | |
| Signaling by SCF-KIT(R) | 0,0267 | 290 | 28 | 2,79E-12 | 3,37E-10 | TP53,BRAF,RET,NEFL,JAK2,EGFR,NRAS,STAT1,STAT3,IRS2,FGF1,FGF2,FGF8,FGF9,HGF,TSC2,RAF1,GDNF,NF1,MDM2,FGFR4,FGFR3,FGFR1,PTEN,NRG1,MMP9,CBL,EGF | |
| MAPK signaling pathway(K) | 0,0235 | 255 | 26 | 5,47E-12 | 5,64E-10 | TP53,CD14,JUN,BRAF,EGFR,NRAS,NTF3,FAS,FGF1,FGF2,TNF,FGF8,FGF9,RAF1,BDNF,NF1,FGFR4,FGFR3,FGFR1,HSPB1,TRAF2,NGF,TRAF6,NTRK1,NTRK2,EGF | |
| Direct p53 effectors(N) | 0,0121 | 132 | 19 | 1,62E-11 | 1,45E-09 | TP63,TP53,ATF3,JUN,LIF,PCNA,EGFR,SPP1,FAS,HGF,TSC2,VDR,SP1,MDM2,PTEN,MMP2,HDAC2,E2F1,BAX | |
| Signaling by EGFR(R) | 0,0292 | 317 | 28 | 2,18E-11 | 1,74E-09 | TP53,BRAF,RET,NEFL,JAK2,EGFR,NRAS,IRS2,FGF1,FGF2,FGF8,FGF9,HGF,ADAM10,TSC2,ADAM17,ADAM12,RAF1,GDNF,NF1,MDM2,FGFR4,FGFR3,FGFR1,PTEN,NRG1,CBL,EGF | |
| EGFR tyrosine kinase inhibitor resistance(K) | 0,0075 | 81 | 15 | 7,94E-11 | 5,72E-09 | BRAF,IGF1,JAK2,EGFR,NRAS,STAT3,FGF2,HGF,RAF1,NF1,FGFR3,PTEN,NRG1,EGF,BAX | |
| Hepatitis B(K) | 0,0134 | 146 | 19 | 8,70E-11 | 5,74E-09 | TP53,FADD,JUN,NFATC4,TLR4,TLR3,TLR2,PCNA,NRAS,STAT1,STAT2,STAT3,FAS,TNF,RAF1,PTEN,MMP9,E2F1,BAX | |
| p75(NTR)-mediated signaling(N) | 0,0063 | 69 | 14 | 1,07E-10 | 6,42E-09 | APP,TP53,NGFR,XIAP,NTF3,MAG,ADAM17,RTN4,BDNF,MMP3,NGF,TRAF6,E2F1,NTRK1 | |
| Signaling by PDGF(R) | 0,0302 | 328 | 27 | 2,39E-10 | 1,31E-08 | TP53,BRAF,RET,NEFL,JAK2,EGFR,NRAS,SPP1,STAT1,STAT3,IRS2,FGF1,FGF2,FGF8,FGF9,HGF,TSC2,RAF1,GDNF,NF1,MDM2,FGFR4,FGFR3,FGFR1,PTEN,NRG1,EGF | |
| Signaling pathways regulating pluripotency of stem cells(K) | 0,0131 | 142 | 18 | 4,20E-10 | 2,14E-08 | SOX2,ISL1,LIF,IGF1,NANOG,JAK2,PAX6,NRAS,STAT3,FGF2,RAF1,BMP4,BMP2,FGFR4,FGFR3,FGFR1,KLF4,REST | |
| Bladder cancer(K) | 0,0038 | 41 | 11 | 6,40E-10 | 2,81E-08 | TP53,BRAF,EGFR,NRAS,RAF1,MDM2,FGFR3,MMP2,MMP9,E2F1,EGF | |
| Breast cancer(K) | 0,0134 | 146 | 18 | 6,48E-10 | 2,81E-08 | TP53,JUN,BRAF,IGF1,EGFR,NRAS,FGF1,FGF2,FGF8,FGF9,RAF1,NOTCH3,SP1,FGFR1,PTEN,E2F1,HES1,EGF | |
| PIP3 activates AKT signaling(R) | 0,0102 | 111 | 16 | 6,69E-10 | 2,81E-08 | TP53,EGFR,IRS2,FGF1,FGF2,FGF8,FGF9,HGF,TSC2,MDM2,FGFR4,FGFR3,FGFR1,PTEN,NRG1,EGF | |
| Signaling by Leptin(R) | 0,0192 | 209 | 21 | 8,81E-10 | 3,53E-08 | BRAF,RET,NEFL,JAK2,EGFR,NRAS,STAT3,IRS2,FGF1,FGF2,FGF8,FGF9,HGF,RAF1,GDNF,NF1,FGFR4,FGFR3,FGFR1,NRG1,EGF | |
| RAF/MAP kinase cascade(R) | 0,0185 | 201 | 20 | 2,67E-09 | 1,01E-07 | BRAF,RET,NEFL,JAK2,EGFR,NRAS,IRS2,FGF1,FGF2,FGF8,FGF9,HGF,RAF1,GDNF,NF1,FGFR4,FGFR3,FGFR1,NRG1,EGF | |
| Signaling by Type 1 Insulin-like Growth Factor 1 Receptor (IGF1R)(R) | 0,0235 | 255 | 22 | 5,40E-09 | 1,94E-07 | BRAF,IGF1,RET,NEFL,JAK2,EGFR,NRAS,IRS2,FGF1,FGF2,FGF8,FGF9,HGF,TSC2,RAF1,GDNF,NF1,FGFR4,FGFR3,FGFR1,NRG1,EGF | |
| Endocrine resistance(K) | 0,009 | 98 | 14 | 8,99E-09 | 3,06E-07 | TP53,JUN,BRAF,IGF1,EGFR,NRAS,RAF1,NOTCH3,SP1,MDM2,MMP2,MMP9,E2F1,BAX | |
| RET signaling(R) | 0,0204 | 222 | 20 | 1,37E-08 | 4,54E-07 | BRAF,RET,NEFL,JAK2,EGFR,NRAS,IRS2,FGF1,FGF2,FGF8,FGF9,HGF,RAF1,GDNF,NF1,FGFR4,FGFR3,FGFR1,NRG1,EGF | |
| DAP12 interactions(R) | 0,0297 | 323 | 24 | 1,79E-08 | 5,55E-07 | TP53,BRAF,RET,NEFL,JAK2,EGFR,NRAS,IRS2,FGF1,FGF2,FGF8,FGF9,HGF,TSC2,RAF1,GDNF,NF1,MDM2,FGFR4,FGFR3,FGFR1,PTEN,NRG1,EGF | |
| NCAM signaling for neurite out-growth(R) | 0,0213 | 231 | 20 | 2,62E-08 | 7,87E-07 | BRAF,RET,NEFL,JAK2,EGFR,NRAS,IRS2,FGF1,FGF2,FGF8,FGF9,HGF,RAF1,GDNF,NF1,FGFR4,FGFR3,FGFR1,NRG1,EGF | |
| FGF signaling pathway(N) | 0,0043 | 47 | 10 | 3,37E-08 | 9,78E-07 | JUN,SPP1,STAT1,FGF1,CDH2,FGFR4,FGFR3,FGFR1,MMP9,CBL | |
| PI3K-Akt signaling pathway(K) | 0,0314 | 341 | 24 | 4,89E-08 | 1,32E-06 | TP53,NGFR,IGF1,JAK2,TLR4,TLR2,EGFR,NRAS,SPP1,FGF1,FGF2,FGF8,FGF9,HGF,TSC2,RAF1,GNB4,MDM2,FGFR4,FGFR3,FGFR1,PTEN,NGF,EGF | |
| Gastrin-CREB signalling pathway via PKC and MAPK(R) | 0,0346 | 376 | 25 | 7,41E-08 | 1,93E-06 | APP,BRAF,RET,NEFL,JAK2,AVP,EGFR,NRAS,IRS2,FGF1,FGF2,FGF8,FGF9,HGF,RAF1,GDNF,NF1,FGFR4,FGFR3,FGFR1,TRPC6,MMP3,NRG1,EGF,CCK | |
| Fc epsilon receptor (FCERI) signaling(R) | 0,0373 | 405 | 26 | 7,87E-08 | 1,97E-06 | TP53,JUN,BRAF,RET,NEFL,JAK2,EGFR,NRAS,IRS2,FGF1,FGF2,FGF8,FGF9,HGF,TSC2,RAF1,GDNF,NF1,MDM2,FGFR4,FGFR3,FGFR1,PTEN,NRG1,TRAF6,EGF | |
| Central carbon metabolism in cancer(K) | 0,0062 | 67 | 11 | 9,25E-08 | 2,31E-06 | PDK1,TP53,RET,EGFR,NRAS,RAF1,FGFR3,FGFR1,PTEN,NTRK1,NTRK3 | |
| Signaling by Insulin receptor(R) | 0,0255 | 277 | 21 | 1,06E-07 | 2,55E-06 | BRAF,RET,NEFL,JAK2,EGFR,NRAS,IRS2,FGF1,FGF2,FGF8,FGF9,HGF,TSC2,RAF1,GDNF,NF1,FGFR4,FGFR3,FGFR1,NRG1,EGF | |
| Neurotrophin signaling pathway(K) | 0,0111 | 121 | 14 | 1,18E-07 | 2,70E-06 | TP53,NGFR,JUN,BRAF,NRAS,NTF3,RAF1,BDNF,NGF,TRAF6,NTRK1,NTRK2,NTRK3,BAX | |
| Signaling by VEGF(R) | 0,0264 | 287 | 21 | 1,90E-07 | 4,17E-06 | BRAF,RET,NEFL,JAK2,EGFR,NRAS,IRS2,FGF1,FGF2,FGF8,FGF9,HGF,RAF1,GDNF,NF1,FGFR4,FGFR3,FGFR1,HSPB1,NRG1,EGF | |
| Prostate cancer(K) | 0,0082 | 89 | 12 | 1,95E-07 | 4,29E-06 | AR,TP53,BRAF,IGF1,EGFR,NRAS,RAF1,MDM2,FGFR1,PTEN,E2F1,EGF | |
| Neurotrophic factor-mediated Trk receptor signaling(N) | 0,0055 | 60 | 10 | 3,13E-07 | 6,58E-06 | NGFR,NRAS,NTF3,STAT3,FAIM,BDNF,NGF,NTRK1,NTRK2,NTRK3 | |
| Hepatitis C(K) | 0,0122 | 133 | 14 | 3,62E-07 | 7,24E-06 | TP53,BRAF,TLR3,EGFR,NRAS,LDLR,STAT1,STAT2,STAT3,TNF,RAF1,TRAF2,TRAF6,EGF | |
| Glucocorticoid receptor regulatory network(N) | 0,0072 | 78 | 11 | 4,11E-07 | 8,22E-06 | TP53,EGR1,JUN,STAT1,FKBP4,FKBP5,CDK5,MDM2,TBP,HDAC2,BAX | |
| Posttranslational regulation of adherens junction stability and dissassembly(N) | 0,0044 | 48 | 9 | 4,70E-07 | 8,94E-06 | RET,EGFR,CDH2,ADAM10,BDNF,GDNF,MMP3,NTRK2,EGF | |
| Transcriptional misregulation in cancer(K) | 0,0166 | 180 | 16 | 4,87E-07 | 9,25E-06 | TP53,CD14,NGFR,IGF1,CD40,PAX3,ZEB1,MEN1,MYCN,SP1,MDM2,MMP3,MMP9,ETV5,HDAC2,NTRK1 | |
| Validated transcriptional targets of TAp63 isoforms(N) | 0,0045 | 49 | 9 | 5,57E-07 | 1,00E-05 | TP63,SHH,NQO1,FAS,VDR,SP1,MDM2,TRAF4,BAX | |
| L1CAM interactions(R) | 0,0075 | 82 | 11 | 6,67E-07 | 1,20E-05 | ALCAM,SCN1B,L1CAM,CD24,AP2A1,CHL1,EGFR,AP2B1,FGFR1,GAP43,DCX | |
| ErbB2/ErbB3 signaling events(N) | 0,0034 | 37 | 8 | 7,22E-07 | 1,23E-05 | JUN,NFATC4,JAK2,NRAS,STAT3,RAF1,NF2,NRG1 | |
| Glioma(K) | 0,0061 | 66 | 10 | 7,36E-07 | 1,25E-05 | TP53,BRAF,IGF1,EGFR,NRAS,RAF1,MDM2,PTEN,E2F1,EGF | |
| Rap1 signaling pathway(K) | 0,0195 | 212 | 17 | 8,63E-07 | 1,38E-05 | PFN1,NGFR,BRAF,IGF1,EGFR,NRAS,FGF1,FGF2,FGF8,FGF9,HGF,RAF1,FGFR4,FGFR3,FGFR1,NGF,EGF | |
| Ligand-dependent caspase activation(R) | 0,0016 | 17 | 6 | 1,14E-06 | 1,82E-05 | FADD,CD14,TLR4,TLR3,FAS,TRAF2 | |
| Signaling by FGFR3(R) | 0,0037 | 40 | 8 | 1,28E-06 | 1,98E-05 | BRAF,NRAS,FGF1,FGF2,FGF8,FGF9,FGFR3,CBL | |
| Signaling by the B Cell Receptor (BCR)(R) | 0,0248 | 270 | 19 | 1,32E-06 | 1,98E-05 | TP53,EGFR,NRAS,IRS2,FGF1,FGF2,FGF8,FGF9,HGF,TSC2,MDM2,FGFR4,FGFR3,FGFR1,PTEN,TRPC1,NRG1,CBL,EGF | |
| Signaling by FGFR4(R) | 0,0038 | 41 | 8 | 1,54E-06 | 2,31E-05 | BRAF,NRAS,FGF1,FGF2,FGF8,FGF9,FGFR4,CBL | |
| Ras signaling pathway(K) | 0,0211 | 229 | 17 | 2,42E-06 | 3,62E-05 | NGFR,IGF1,EGFR,NRAS,FGF1,FGF2,FGF8,FGF9,HGF,RAF1,GNB4,NF1,FGFR4,FGFR3,FGFR1,NGF,EGF | |
| Herpes simplex infection(K) | 0,017 | 185 | 15 | 3,40E-06 | 4,75E-05 | CLOCK,TP53,FADD,JUN,JAK2,TLR3,TLR2,CD74,STAT1,STAT2,FAS,TNF,TBP,TRAF2,TRAF6 | |
| Toll-Like Receptors Cascades(R) | 0,013 | 141 | 13 | 4,00E-06 | 5,60E-05 | APP,FADD,CD14,JUN,CD36,TLR4,TLR3,TLR2,HMGB1,TRAF6,NOD1,NOD2,S100B | |
| Trk receptor signaling mediated by the MAPK pathway(N) | 0,003 | 33 | 7 | 4,09E-06 | 5,72E-05 | EGR1,BRAF,NRAS,NTF3,RAF1,TRPV1,CDK5 | |
| Regulation of actin cytoskeleton(K) | 0,0197 | 214 | 16 | 4,41E-06 | 5,74E-05 | PFN1,CD14,BRAF,GIT1,EGFR,NRAS,FGF1,FGF2,FGF8,FGF9,RAF1,FGFR4,FGFR3,FGFR1,EGF,LIMK1 | |
| Signaling by FGFR1(R) | 0,0045 | 49 | 8 | 5,63E-06 | 7,31E-05 | BRAF,NRAS,FGF1,FGF2,FGF8,FGF9,FGFR1,CBL | |
| neuroregulin receptor degredation protein-1 controls erbb3 receptor recycling(B) | 0,0006 | 6 | 4 | 6,31E-06 | 8,21E-05 | JAK2,EGFR,NRG1,NRG3 | |
| Toll-like receptor signaling pathway(K) | 0,0098 | 106 | 11 | 7,57E-06 | 9,68E-05 | FADD,CD14,JUN,CD40,TLR4,TLR3,TLR2,SPP1,STAT1,TNF,TRAF6 | |
| p53 pathway feedback loops 2(P) | 0,0022 | 24 | 6 | 8,07E-06 | 9,68E-05 | TP63,TP53,NRAS,STAT1,MDM2,PTEN | |
| Endometrial cancer(K) | 0,0048 | 52 | 8 | 8,62E-06 | 1,03E-04 | TP53,BRAF,EGFR,NRAS,RAF1,PTEN,ILK,EGF | |
| Extracellular matrix organization(R) | 0,0235 | 255 | 17 | 9,76E-06 | 1,17E-04 | DMP1,COL8A2,COL8A1,MMP13,CD47,CD44,SPP1,FGF2,EFEMP1,ADAM10,ADAM17,BMP7,BMP4,BMP2,MMP2,MMP3,MMP9 | |
| AP-1 transcription factor network(N) | 0,0064 | 70 | 9 | 9,80E-06 | 1,18E-04 | DMP1,TP53,ATF3,EGR1,JUN,TH,SP1,PTEN,MMP9 | |
| cbl mediated ligand-induced downregulation of egf receptors pathway(B) | 0,0006 | 7 | 4 | 1,15E-05 | 1,38E-04 | JAK2,EGFR,CBL,EGF | |
| FoxO signaling pathway(K) | 0,0123 | 134 | 12 | 1,26E-05 | 1,38E-04 | FOXG1,BRAF,IGF1,EGFR,NRAS,STAT3,IRS2,RAF1,MDM2,PTEN,CAT,EGF | |
| Signaling by ERBB4(R) | 0,0039 | 42 | 7 | 1,92E-05 | 2,11E-04 | EGFR,NRAS,ADAM17,GFAP,NRG1,EGF,S100B | |
| Apoptosis(K) | 0,0129 | 140 | 12 | 1,93E-05 | 2,12E-04 | TP53,FADD,JUN,XIAP,NRAS,FAS,TNF,RAF1,TRAF2,NGF,NTRK1,BAX | |
| Osteopontin-mediated events(N) | 0,0027 | 29 | 6 | 2,32E-05 | 2,55E-04 | JUN,CD44,SPP1,ILK,MMP2,MMP9 | |
| Notch-mediated HES/HEY network(N) | 0,0043 | 47 | 7 | 3,89E-05 | 4,24E-04 | AR,TWIST1,JAK2,STAT3,YY1,E2F1,HES1 | |
| toll-like receptor pathway(B) | 0,0029 | 32 | 6 | 4,00E-05 | 4,24E-04 | CD14,JUN,TLR4,TLR3,TLR2,TRAF6 | |
| Axon guidance(K) | 0,0163 | 177 | 13 | 4,24E-05 | 4,24E-04 | PDK1,L1CAM,NFATC4,SHH,NRAS,RAF1,BMP7,CDK5,ILK,TRPC5,TRPC6,TRPC1,LIMK1 | |
| Ceramide signaling pathway(N) | 0,0044 | 48 | 7 | 4,43E-05 | 4,43E-04 | FADD,IGF1,TNF,RAF1,TRAF2,EGF,BAX | |
| Pancreatic cancer(K) | 0,0061 | 66 | 8 | 4,63E-05 | 4,63E-04 | TP53,BRAF,EGFR,STAT1,STAT3,RAF1,E2F1,EGF | |
| Endocytosis(K) | 0,0239 | 260 | 16 | 4,64E-05 | 4,64E-04 | RET,AP2A1,GIT1,EGFR,CXCR1,CXCR2,LDLR,GIT2,AP2B1,MDM2,FGFR4,FGFR3,TRAF6,CBL,NTRK1,EGF | |
| Tuberculosis(K) | 0,0165 | 179 | 13 | 4,75E-05 | 4,75E-04 | FADD,CD14,JAK2,TLR4,TLR2,CD74,STAT1,TNF,RAF1,VDR,TRAF6,NOD2,BAX | |
| Malaria(K) | 0,0045 | 49 | 7 | 5,04E-05 | 5,04E-04 | CD40,CD36,TLR4,TLR2,TNF,HGF,LRP1 | |
| Epithelial cell signaling in Helicobacter pylori infection(K) | 0,0063 | 68 | 8 | 5,69E-05 | 5,13E-04 | JUN,GIT1,EGFR,CXCR1,CXCR2,ADAM10,ADAM17,NOD1 | |
| ErbB signaling pathway(K) | 0,0081 | 88 | 9 | 5,74E-05 | 5,16E-04 | JUN,BRAF,EGFR,NRAS,RAF1,NRG1,NRG3,CBL,EGF | |
| erk1/erk2 mapk signaling pathway(B) | 0,0019 | 21 | 5 | 5,88E-05 | 5,29E-04 | NGFR,JAK2,STAT3,RAF1,NGF | |
| SHP2 signaling(N) | 0,0047 | 51 | 7 | 6,46E-05 | 5,81E-04 | IGF1,JAK2,EGFR,NRAS,STAT1,RAF1,EGF | |
| Caspase cascade in apoptosis(N) | 0,0048 | 52 | 7 | 7,28E-05 | 6,55E-04 | APP,XIAP,TNF,TFAP2A,TRAF2,LIMK1,BAX | |
| FGF signaling pathway(P) | 0,0085 | 92 | 9 | 8,02E-05 | 7,22E-04 | NRAS,FGF1,FGF2,FGF8,FGF9,RAF1,FGFR4,FGFR3,FGFR1 | |
| role of erk5 in neuronal survival pathway(B) | 0,0021 | 23 | 5 | 8,99E-05 | 8,09E-04 | JAK2,NTF3,RAF1,BDNF,NGF | |
| Chronic myeloid leukemia(K) | 0,0067 | 73 | 8 | 9,27E-05 | 8,34E-04 | TP53,BRAF,NRAS,RAF1,MDM2,HDAC2,CBL,E2F1 | |
| Toxoplasmosis(K) | 0,0109 | 118 | 10 | 1,05E-04 | 8,39E-04 | CD40,JAK2,TLR4,TLR2,XIAP,LDLR,STAT1,STAT3,TNF,TRAF6 | |
| Thyroid hormone signaling pathway(K) | 0,0109 | 118 | 10 | 1,05E-04 | 8,39E-04 | TP53,NRAS,STAT1,TSC2,RAF1,NOTCH3,BMP4,MDM2,HDAC2,ATP1A1 | |
| Growth hormone receptor signaling(R) | 0,0022 | 24 | 5 | 1,09E-04 | 8,76E-04 | JAK2,STAT1,STAT3,IRS2,ADAM17 | |
| Non-small cell lung cancer(K) | 0,0052 | 56 | 7 | 1,15E-04 | 9,18E-04 | TP53,BRAF,EGFR,NRAS,RAF1,E2F1,EGF | |
| PDGFR-beta signaling pathway(N) | 0,011 | 120 | 10 | 1,20E-04 | 9,61E-04 | JUN,BRAF,JAK2,NRAS,STAT1,STAT3,RAF1,PTEN,LRP1,CBL | |
| Endogenous TLR signaling(N) | 0,0023 | 25 | 5 | 1,32E-04 | 1,06E-03 | CD14,TLR4,TLR3,TLR2,HMGB1 | |
| tpo signaling pathway(B) | 0,0023 | 25 | 5 | 1,32E-04 | 1,06E-03 | JUN,JAK2,STAT1,STAT3,RAF1 | |
| ErbB1 downstream signaling(N) | 0,0092 | 100 | 9 | 1,49E-04 | 1,11E-03 | EGR1,JUN,BRAF,EGFR,NRAS,STAT1,STAT3,RAF1,EGF | |
| Estrogen signaling pathway(K) | 0,0092 | 100 | 9 | 1,49E-04 | 1,11E-03 | JUN,EGFR,NRAS,FKBP4,RAF1,FKBP5,SP1,MMP2,MMP9 | |
| pdgf signaling pathway(B) | 0,0024 | 26 | 5 | 1,58E-04 | 1,11E-03 | JUN,JAK2,STAT1,STAT3,RAF1 | |
| IL27-mediated signaling events(N) | 0,0024 | 26 | 5 | 1,58E-04 | 1,11E-03 | JAK2,STAT1,STAT2,STAT3,TNF | |
| AGE-RAGE signaling pathway in diabetic complications(K) | 0,0093 | 101 | 9 | 1,61E-04 | 1,12E-03 | EGR1,JUN,JAK2,NRAS,STAT1,STAT3,TNF,MMP2,BAX | |
| Urokinase-type plasminogen activator (uPA) and uPAR-mediated signaling(N) | 0,0039 | 42 | 6 | 1,75E-04 | 1,22E-03 | MMP13,EGFR,HGF,MMP3,MMP9,LRP1 | |
| Signaling by PTK6(R) | 0,0056 | 61 | 7 | 1,93E-04 | 1,35E-03 | EGFR,NRAS,STAT3,NF1,NRG1,CBL,EGF | |
| Cytokine-cytokine receptor interaction(K) | 0,0244 | 265 | 15 | 1,98E-04 | 1,38E-03 | CNTF,NGFR,LIF,CD27,CD40,EGFR,CXCR1,CXCR2,FAS,TNF,HGF,BMP7,BMP2,AMH,EGF | |
| Generic Transcription Pathway(R) | 0,0455 | 494 | 22 | 2,14E-04 | 1,50E-03 | TP63,AR,TP53,JUN,PCNA,EGFR,FAS,YY1,MEN1,TSC2,NOTCH3,TFAP2A,VDR,CDK5,SP1,MDM2,PTEN,TBP,HDAC2,E2F1,E2F4,BAX | |
| ErbB receptor signaling network(N) | 0,0014 | 15 | 4 | 2,16E-04 | 1,51E-03 | EGFR,NRG1,NRG3,EGF | |
| p53 pathway(P) | 0,004 | 44 | 6 | 2,24E-04 | 1,57E-03 | TP63,TP53,FAS,MDM2,PTEN,BAX | |
| P53 pathway feedback loops 1(P) | 0,0006 | 6 | 3 | 2,25E-04 | 1,58E-03 | TP63,TP53,MDM2 | |
| Response to elevated platelet cytosolic Ca2+(R) | 0,0098 | 106 | 9 | 2,29E-04 | 1,60E-03 | APP,PFN1,IGF1,CD36,CD63,APLP2,HGF,EGF,SOD1 | |
| Validated transcriptional targets of deltaNp63 isoforms(N) | 0,0041 | 45 | 6 | 2,52E-04 | 1,65E-03 | TP63,FASN,VDR,MDM2,NRG1,HES1 | |
| EGF receptor (ErbB1) signaling pathway(N) | 0,0027 | 29 | 5 | 2,61E-04 | 1,65E-03 | EGFR,NRAS,STAT1,STAT3,EGF | |
| Thyroid cancer(K) | 0,0027 | 29 | 5 | 2,61E-04 | 1,65E-03 | TP53,BRAF,RET,NRAS,NTRK1 | |
| nerve growth factor pathway (ngf)(B) | 0,0015 | 16 | 4 | 2,75E-04 | 1,65E-03 | NGFR,JUN,RAF1,NGF | |
| Inflammatory bowel disease (IBD)(K) | 0,006 | 65 | 7 | 2,82E-04 | 1,69E-03 | JUN,TLR4,TLR2,STAT1,STAT3,TNF,NOD2 | |
| TNF signaling pathway(K) | 0,0101 | 110 | 9 | 2,99E-04 | 1,79E-03 | FADD,JUN,LIF,FAS,TNF,MMP3,TRAF2,MMP9,NOD2 | |
| ceramide signaling pathway(B) | 0,0028 | 30 | 5 | 3,05E-04 | 1,83E-03 | FADD,TNF,RAF1,TRAF2,BAX | |
| Alzheimer disease-presenilin pathway(P) | 0,0102 | 111 | 9 | 3,19E-04 | 1,92E-03 | APP,BACE1,MMP13,CD44,ADAM17,NOTCH3,MMP2,MMP9,LRP1 | |
| amb2 Integrin signaling(N) | 0,0029 | 31 | 5 | 3,54E-04 | 2,12E-03 | TNF,HMGB1,MMP2,MMP9,LRP1 | |
| p53 signaling pathway(K) | 0,0063 | 69 | 7 | 4,02E-04 | 2,41E-03 | TP53,IGF1,FAS,TSC2,MDM2,PTEN,BAX | |
| Adipocytokine signaling pathway(K) | 0,0064 | 70 | 7 | 4,38E-04 | 2,63E-03 | CD36,JAK2,NPY,STAT3,IRS2,TNF,TRAF2 | |
| Signaling by FGFR2(R) | 0,0065 | 71 | 7 | 4,76E-04 | 2,86E-03 | BRAF,NRAS,FGF1,FGF2,FGF8,FGF9,CBL | |
| NOD-like receptor signaling pathway(K) | 0,0156 | 170 | 11 | 4,78E-04 | 2,87E-03 | FADD,JUN,TLR4,XIAP,STAT1,STAT2,TNF,TRAF2,TRAF6,NOD1,NOD2 | |
| Amyotrophic lateral sclerosis (ALS)(K) | 0,0047 | 51 | 6 | 4,86E-04 | 2,92E-03 | TP53,NEFL,TNF,CAT,SOD1,BAX | |
| HTLV-I infection(K) | 0,0237 | 258 | 14 | 4,88E-04 | 2,93E-03 | TP53,ATF3,EGR1,JUN,NFATC4,CD40,PCNA,XIAP,NRAS,MSX1,TNF,TBP,E2F1,BAX | |
| ifn alpha signaling pathway(B) | 0,0007 | 8 | 3 | 5,20E-04 | 3,05E-03 | JAK2,STAT1,STAT2 | |
| sprouty regulation of tyrosine kinase signals(B) | 0,0017 | 19 | 4 | 5,24E-04 | 3,05E-03 | JAK2,RAF1,CBL,EGF | |
| Signaling events mediated by Stem cell factor receptor (c-Kit)(N) | 0,0048 | 52 | 6 | 5,38E-04 | 3,05E-03 | JAK2,STAT1,STAT3,RAF1,PTEN,CBL | |
| Leishmaniasis(K) | 0,0067 | 73 | 7 | 5,60E-04 | 3,05E-03 | JUN,JAK2,TLR4,TLR2,STAT1,TNF,TRAF6 | |
| Huntington disease(P) | 0,0111 | 121 | 9 | 5,90E-04 | 3,05E-03 | TP63,TP53,JUN,AP2A1,HAP1,TUBB3,BDNF,TBP,BAX | |
| Internalization of ErbB1(N) | 0,0032 | 35 | 5 | 6,10E-04 | 3,05E-03 | EGFR,NRAS,RAF1,CBL,EGF | |
| Signaling events mediated by TCPTP(N) | 0,0032 | 35 | 5 | 6,10E-04 | 3,05E-03 | EGFR,STAT1,STAT3,HGF,EGF | |
| IL2-mediated signaling events(N) | 0,005 | 54 | 6 | 6,54E-04 | 3,27E-03 | JUN,NRAS,STAT1,STAT3,IRS2,RAF1 | |
| p73 transcription factor network(N) | 0,0069 | 75 | 7 | 6,56E-04 | 3,28E-03 | TP63,FASN,FAS,SP1,MDM2,NTRK1,BAX | |
| MicroRNAs in cancer(K) | 0,0275 | 299 | 15 | 6,89E-04 | 3,44E-03 | TP63,TP53,ZEB1,CD44,EGFR,NRAS,STAT3,IRS2,RAF1,NOTCH3,MDM2,FGFR3,PTEN,MMP9,E2F1 | |
| Trk receptor signaling mediated by PI3K and PLC-gamma(N) | 0,0033 | 36 | 5 | 6,92E-04 | 3,46E-03 | EGR1,NRAS,TRPV1,NGF,NTRK1 | |
| Transcriptional regulation of pluripotent stem cells(R) | 0,0033 | 36 | 5 | 6,92E-04 | 3,46E-03 | SOX2,NANOG,STAT3,FGF2,KLF4 | |
| egf signaling pathway(B) | 0,0019 | 21 | 4 | 7,59E-04 | 3,79E-03 | JAK2,STAT1,STAT3,EGF | |
| nf-kb signaling pathway(B) | 0,0019 | 21 | 4 | 7,59E-04 | 3,79E-03 | FADD,TLR4,TNF,TRAF6 | |
| p38 signaling mediated by MAPKAP kinases(N) | 0,0019 | 21 | 4 | 7,59E-04 | 3,79E-03 | TH,TSC2,RAF1,HSPB1 | |
| Regulation of nuclear SMAD2/3 signaling(N) | 0,0071 | 77 | 7 | 7,64E-04 | 3,82E-03 | AR,FOXG1,JUN,DLX1,VDR,SP1,HDAC2 | |
| Validated transcriptional targets of AP1 family members Fra1 and Fra2(N) | 0,0034 | 37 | 5 | 7,81E-04 | 3,91E-03 | JUN,LIF,SP1,MMP2,MMP9 | |
| GMCSF-mediated signaling events(N) | 0,0034 | 37 | 5 | 7,81E-04 | 3,91E-03 | JAK2,NRAS,STAT1,STAT3,RAF1 | |
| inhibition of cellular proliferation by gleevec(B) | 0,002 | 22 | 4 | 9,00E-04 | 4,50E-03 | JUN,JAK2,STAT1,RAF1 | |
| ATF-2 transcription factor network(N) | 0,0053 | 58 | 6 | 9,43E-04 | 4,71E-03 | ATF3,JUN,TH,NF1,MMP2,HES1 | |
| g-secretase mediated erbb4 signaling pathway(B) | 0,0009 | 10 | 3 | 9,87E-04 | 4,93E-03 | JAK2,ADAM17,NRG3 | |
| il22 soluble receptor signaling pathway(B) | 0,0009 | 10 | 3 | 9,87E-04 | 4,93E-03 | JAK2,STAT1,STAT3 | |
| EGF receptor signaling pathway(P) | 0,0075 | 82 | 7 | 1,10E-03 | 5,48E-03 | EGFR,NRAS,STAT1,STAT3,RAF1,NF1,CBL | |
| Intrinsic Pathway for Apoptosis(R) | 0,0038 | 41 | 5 | 1,23E-03 | 5,74E-03 | TP63,TP53,XIAP,E2F1,BAX | |
| TGF-beta signaling pathway(K) | 0,0077 | 84 | 7 | 1,26E-03 | 5,74E-03 | TNF,BMP7,BMP4,BMP2,SP1,AMH,E2F4 | |
| Huntington's disease(K) | 0,0178 | 193 | 11 | 1,33E-03 | 5,74E-03 | TP53,AP2A1,HAP1,AP2B1,BDNF,SP1,TBP,HDAC2,SOD1,REST,BAX | |
| Measles(K) | 0,0125 | 136 | 9 | 1,33E-03 | 5,74E-03 | TP53,JAK2,TLR4,TLR2,STAT1,STAT2,STAT3,FAS,TRAF6 | |
| Stabilization and expansion of the E-cadherin adherens junction(N) | 0,0039 | 42 | 5 | 1,36E-03 | 5,74E-03 | IGF1,GIT1,EGFR,HGF,EGF | |
| FOXA1 transcription factor network(N) | 0,0039 | 42 | 5 | 1,36E-03 | 5,74E-03 | AR,JUN,SHH,SP1,SOD1 | |
| Ras Pathway(P) | 0,0058 | 63 | 6 | 1,43E-03 | 5,74E-03 | JUN,BRAF,NRAS,STAT1,STAT3,RAF1 | |
| Glypican 1 network(N) | 0,0023 | 25 | 4 | 1,44E-03 | 5,74E-03 | APP,FGF2,FGFR1,NRG1 | |
| Small cell lung cancer(K) | 0,0079 | 86 | 7 | 1,44E-03 | 5,75E-03 | TP53,XIAP,PTEN,TRAF2,TRAF4,TRAF6,E2F1 | |
| Neutrophil degranulation(R) | 0,0362 | 393 | 17 | 1,54E-03 | 6,17E-03 | MIF,CD14,CD36,CD47,CD44,TLR2,CD63,NRAS,CXCR1,CXCR2,CD68,ADAM10,HPSE,HMGB1,MMP9,CAT,PTX3 | |
| Downstream signaling in na&#xef;ve CD8+ T cells(N) | 0,0059 | 64 | 6 | 1,55E-03 | 6,20E-03 | EGR1,JUN,BRAF,NRAS,TNF,RAF1 | |
| Longevity regulating pathway - multiple species(K) | 0,0059 | 64 | 6 | 1,55E-03 | 6,20E-03 | IGF1,NRAS,IRS2,CAT,HDAC2,SOD1 | |
| Regulation of retinoblastoma protein(N) | 0,0059 | 64 | 6 | 1,55E-03 | 6,20E-03 | JUN,PAX3,RAF1,TBP,E2F1,E2F4 | |
| Ion channel transport(R) | 0,0156 | 169 | 10 | 1,66E-03 | 6,64E-03 | WNK1,WNK3,RAF1,ASIC3,TRPV1,TRPC5,TRPC6,TRPC1,ATP1A1,TRPA1 | |
| EPH-Ephrin signaling(R) | 0,0082 | 89 | 7 | 1,74E-03 | 6,97E-03 | AP2A1,GIT1,AP2B1,ADAM10,MMP2,MMP9,LIMK1 | |
| Angiogenesis(P) | 0,0061 | 66 | 6 | 1,81E-03 | 7,23E-03 | JUN,NRAS,STAT1,STAT3,FGF1,FGFR1 | |
| Alzheimer's disease(K) | 0,0157 | 171 | 10 | 1,81E-03 | 7,23E-03 | APP,FADD,BACE1,FAS,TNF,ADAM10,ADAM17,RTN4,CDK5,LRP1 | |
| Focal adhesion(K) | 0,0185 | 201 | 11 | 1,82E-03 | 7,27E-03 | JUN,BRAF,IGF1,XIAP,EGFR,SPP1,HGF,RAF1,PTEN,ILK,EGF | |
| TNF receptor signaling pathway(N) | 0,0042 | 46 | 5 | 2,02E-03 | 8,09E-03 | FADD,STAT1,TNF,ADAM17,TRAF2 | |
| Epstein-Barr virus infection(K) | 0,0188 | 204 | 11 | 2,04E-03 | 8,15E-03 | TP53,JUN,CD40,CD44,STAT3,MDM2,HSPB1,TBP,TRAF2,TRAF6,HDAC2 | |
| phosphorylation of mek1 by cdk5/p35 down regulates the map kinase pathway(B) | 0,0012 | 13 | 3 | 2,08E-03 | 8,32E-03 | NGFR,RAF1,NGF | |
| cadmium induces dna synthesis and proliferation in macrophages(B) | 0,0012 | 13 | 3 | 2,08E-03 | 8,32E-03 | JUN,TNF,RAF1 | |
| Advanced glycosylation endproduct receptor signaling(R) | 0,0012 | 13 | 3 | 2,08E-03 | 8,32E-03 | APP,HMGB1,S100B | |
| il12 and stat4 dependent signaling pathway in th1 development(B) | 0,0012 | 13 | 3 | 2,08E-03 | 8,32E-03 | JUN,JAK2,ETV5 | |
| Regulation of Telomerase(N) | 0,0063 | 68 | 6 | 2,10E-03 | 8,38E-03 | JUN,EGFR,SP1,HDAC2,E2F1,EGF | |
| role of erbb2 in signal transduction and oncology(B) | 0,0026 | 28 | 4 | 2,16E-03 | 8,65E-03 | JAK2,EGFR,STAT3,RAF1 | |
| ErbB4 signaling events(N) | 0,0026 | 28 | 4 | 2,16E-03 | 8,65E-03 | JAK2,ADAM17,MDM2,NRG1 | |
| Integrins in angiogenesis(N) | 0,0043 | 47 | 5 | 2,22E-03 | 8,88E-03 | IGF1,SPP1,FGF2,ILK,CBL | |
| keratinocyte differentiation(B) | 0,0043 | 47 | 5 | 2,22E-03 | 8,88E-03 | JUN,TNF,RAF1,TRAF2,EGF | |
| Sphingolipid signaling pathway(K) | 0,011 | 120 | 8 | 2,33E-03 | 9,30E-03 | TP53,SPTLC1,NRAS,TNF,RAF1,PTEN,TRAF2,BAX | |
| Longevity regulating pathway(K) | 0,0087 | 94 | 7 | 2,36E-03 | 9,44E-03 | TP53,IGF1,NRAS,IRS2,TSC2,CAT,BAX | |
| CDC42 signaling events(N) | 0,0064 | 70 | 6 | 2,42E-03 | 9,67E-03 | JUN,BRAF,PAX6,RAF1,CBL,LIMK1 | |
| CD40/CD40L signaling(N) | 0,0027 | 29 | 4 | 2,45E-03 | 9,81E-03 | JUN,CD40,TRAF2,TRAF6 | |
| NF-kappa B signaling pathway(K) | 0,0087 | 95 | 7 | 2,50E-03 | 0,01 | CD14,CD40,TLR4,XIAP,TNF,TRAF2,TRAF6 | |
| Ras signaling in the CD4+ TCR pathway(N) | 0,0013 | 14 | 3 | 2,56E-03 | 0,0102 | BRAF,NRAS,RAF1 | |
| Regulation of Androgen receptor activity(N) | 0,0045 | 49 | 5 | 2,65E-03 | 0,0102 | AR,EGR1,JUN,HOXB13,MDM2 | |
| generation of amyloid b-peptide by ps1(B) | 0,0004 | 4 | 2 | 2,75E-03 | 0,0102 | APP,ADAM10 | |
| ifn gamma signaling pathway(B) | 0,0004 | 4 | 2 | 2,75E-03 | 0,0102 | JAK2,STAT1 | |
| Prolactin signaling pathway(K) | 0,0066 | 72 | 6 | 2,78E-03 | 0,0102 | TH,JAK2,NRAS,STAT1,STAT3,RAF1 | |
| calcium signaling by hbx of hepatitis b virus(B) | 0,0014 | 15 | 3 | 3,11E-03 | 0,0102 | JUN,JAK2,RAF1 | |
| Regulated Necrosis(R) | 0,0014 | 15 | 3 | 3,11E-03 | 0,0102 | FADD,FAS,TRAF2 | |
| atm signaling pathway(B) | 0,0014 | 15 | 3 | 3,11E-03 | 0,0102 | JUN,JAK2,MDM2 | |
| role of egf receptor transactivation by gpcrs in cardiac hypertrophy(B) | 0,0029 | 31 | 4 | 3,11E-03 | 0,0102 | JUN,ADAM12,RAF1,EGF | |
| Platinum drug resistance(K) | 0,0069 | 75 | 6 | 3,38E-03 | 0,0102 | TP53,FADD,XIAP,FAS,MDM2,BAX | |
| Signaling events mediated by PTP1B(N) | 0,0048 | 52 | 5 | 3,41E-03 | 0,0102 | JAK2,EGFR,STAT3,CDH2,EGF | |
| Chemokine signaling pathway(K) | 0,0172 | 187 | 10 | 3,41E-03 | 0,0102 | BRAF,JAK2,NRAS,CXCR1,CXCR2,STAT1,STAT2,STAT3,RAF1,GNB4 | |
| Oncogene Induced Senescence(R) | 0,0029 | 32 | 4 | 3,48E-03 | 0,0104 | TP53,SP1,MDM2,E2F1 | |
| Syndecan-4-mediated signaling events(N) | 0,0029 | 32 | 4 | 3,48E-03 | 0,0104 | FGF2,ADAM12,FGFR1,MMP9 | |
| Choline metabolism in cancer(K) | 0,0093 | 101 | 7 | 3,49E-03 | 0,0105 | JUN,EGFR,NRAS,TSC2,RAF1,SP1,EGF | |
| Pertussis(K) | 0,007 | 76 | 6 | 3,61E-03 | 0,0108 | CD14,JUN,TLR4,TNF,TRAF6,NOD1 | |
| RIG-I/MDA5 mediated induction of IFN-alpha/beta pathways(R) | 0,0071 | 77 | 6 | 3,84E-03 | 0,0115 | APP,FADD,HMGB1,TRAF2,TRAF6,S100B | |
| Apoptosis - multiple species(K) | 0,003 | 33 | 4 | 3,88E-03 | 0,0116 | FADD,NGFR,XIAP,BAX | |
| EPO signaling pathway(N) | 0,003 | 33 | 4 | 3,88E-03 | 0,0116 | JAK2,STAT1,IRS2,CBL | |
| HIV-1 Nef: Negative effector of Fas and TNF-alpha(N) | 0,003 | 33 | 4 | 3,88E-03 | 0,0116 | FADD,FAS,TNF,TRAF2 | |
| Chagas disease (American trypanosomiasis)(K) | 0,0096 | 104 | 7 | 4,09E-03 | 0,0123 | FADD,JUN,TLR4,TLR2,FAS,TNF,TRAF6 | |
| Insulin/IGF pathway-protein kinase B signaling cascade(P) | 0,0005 | 5 | 2 | 4,24E-03 | 0,0127 | MDM2,PTEN | |
| FasL/ CD95L signaling(R) | 0,0005 | 5 | 2 | 4,24E-03 | 0,0127 | FADD,FAS | |
| tnfr1 signaling pathway(B) | 0,0016 | 17 | 3 | 4,40E-03 | 0,0132 | FADD,TNF,TRAF2 | |
| Syndecan-1-mediated signaling events(N) | 0,0016 | 17 | 3 | 4,40E-03 | 0,0132 | HGF,HPSE,MMP9 | |
| Regulation of nuclear beta catenin signaling and target gene transcription(N) | 0,0074 | 80 | 6 | 4,61E-03 | 0,0138 | AR,JUN,MMP2,MMP9,HDAC2,KLF4 | |
| CXCR4-mediated signaling events(N) | 0,0074 | 80 | 6 | 4,61E-03 | 0,0138 | JAK2,STAT1,STAT2,STAT3,MMP9,LIMK1 | |
| Arf6 signaling events(N) | 0,0032 | 35 | 4 | 4,76E-03 | 0,0143 | GIT1,EGFR,HGF,EGF | |
| T cell activation(P) | 0,0075 | 81 | 6 | 4,89E-03 | 0,0147 | JUN,BRAF,NFATC4,NRAS,CD74,RAF1 | |
| IL23-mediated signaling events(N) | 0,0034 | 37 | 4 | 5,78E-03 | 0,0173 | JAK2,STAT1,STAT3,TNF | |
| stat3 signaling pathway(B) | 0,0006 | 6 | 2 | 6,03E-03 | 0,0181 | JAK2,STAT3 | |
| cd40l signaling pathway(B) | 0,0006 | 6 | 2 | 6,03E-03 | 0,0181 | CD40,TRAF6 | |
| TNF signaling(R) | 0,0035 | 38 | 4 | 6,34E-03 | 0,019 | FADD,TNF,ADAM17,TRAF2 | |
| Pre-NOTCH Expression and Processing(R) | 0,0035 | 38 | 4 | 6,34E-03 | 0,019 | TP53,JUN,NOTCH3,E2F1 | |
| Nucleotide-binding domain, leucine rich repeat containing receptor (NLR) signaling pathways(R) | 0,0035 | 38 | 4 | 6,34E-03 | 0,019 | APP,TRAF6,NOD1,NOD2 | |
| Phospholipase D signaling pathway(K) | 0,0133 | 144 | 8 | 6,82E-03 | 0,0205 | AVP,EGFR,NRAS,CXCR1,CXCR2,TSC2,RAF1,EGF | |
| Influenza A(K) | 0,0161 | 175 | 9 | 6,85E-03 | 0,0206 | JUN,JAK2,TLR4,TLR3,STAT1,STAT2,FAS,TNF,RAF1 | |
| tnf/stress related signaling(B) | 0,0018 | 20 | 3 | 6,87E-03 | 0,0206 | JUN,TNF,TRAF2 | |
| Signaling events regulated by Ret tyrosine kinase(N) | 0,0036 | 39 | 4 | 6,93E-03 | 0,0208 | JUN,RET,IRS2,GDNF | |
| Colorectal cancer(K) | 0,0057 | 62 | 5 | 7,06E-03 | 0,0212 | TP53,JUN,BRAF,RAF1,BAX | |
| Cell adhesion molecules (CAMs)(K) | 0,0133 | 145 | 8 | 7,10E-03 | 0,0213 | ALCAM,L1CAM,CD40,CD34,CDH2,MPZ,MAG,CNTNAP2 | |
| Clathrin-mediated endocytosis(R) | 0,0108 | 117 | 7 | 7,60E-03 | 0,0228 | AP2A1,AVP,EGFR,LDLR,AP2B1,CBL,EGF | |
| Notch signaling pathway(P) | 0,0019 | 21 | 3 | 7,84E-03 | 0,0235 | ADAM10,NOTCH3,HES1 | |
| mTOR signaling pathway(N) | 0,0059 | 64 | 5 | 8,03E-03 | 0,0241 | BRAF,NRAS,YY1,TSC2,RAF1 | |
| Protein folding(R) | 0,0059 | 64 | 5 | 8,03E-03 | 0,0241 | TP53,CCT5,STAT3,TUBB3,GNB4 | |
| srebp control of lipid synthesis(B) | 0,0006 | 7 | 2 | 8,11E-03 | 0,0243 | SCAP,LDLR | |
| Alcoholism(K) | 0,0166 | 180 | 9 | 8,15E-03 | 0,0244 | BRAF,TH,NRAS,NPY,RAF1,BDNF,GNB4,HDAC2,NTRK2 | |
| BMP receptor signaling(N) | 0,0038 | 41 | 4 | 8,22E-03 | 0,0247 | XIAP,BMP7,BMP4,BMP2 | |
| Plasma membrane estrogen receptor signaling(N) | 0,0039 | 42 | 4 | 8,92E-03 | 0,0261 | IGF1,NRAS,MMP2,MMP9 | |
| Activation of anterior HOX genes in hindbrain development during early embryogenesis(R) | 0,0061 | 66 | 5 | 9,09E-03 | 0,0261 | HOXC4,JUN,PAX6,YY1,WDR5 | |
| Renal cell carcinoma(K) | 0,0062 | 67 | 5 | 9,65E-03 | 0,0261 | JUN,BRAF,NRAS,HGF,RAF1 | |
| Signaling by ERBB2(R) | 0,004 | 43 | 4 | 9,66E-03 | 0,0261 | EGFR,NRAS,NRG1,EGF | |
| Beta3 integrin cell surface interactions(N) | 0,004 | 43 | 4 | 9,66E-03 | 0,0261 | L1CAM,CD47,SPP1,HMGB1 | |
| alk in cardiac myocytes(B) | 0,0021 | 23 | 3 | 0,01 | 0,0261 | BMP7,BMP4,BMP2 | |
| links between pyk2 and map kinases(B) | 0,0021 | 23 | 3 | 0,01 | 0,0261 | JUN,JAK2,RAF1 | |
| Canonical NF-kappaB pathway(N) | 0,0021 | 23 | 3 | 0,01 | 0,0261 | TNF,TRAF6,NOD2 | |
| il-2 receptor beta chain in t cell activation(B) | 0,004 | 44 | 4 | 0,0104 | 0,0261 | JAK2,PCNA,RAF1,CBL | |
| yaci and bcma stimulation of b cell immune responses(B) | 0,0007 | 8 | 2 | 0,0105 | 0,0261 | TRAF2,TRAF6 | |
| tsp-1 induced apoptosis in microvascular endothelial cell(B) | 0,0007 | 8 | 2 | 0,0105 | 0,0261 | JUN,JAK2 | |
| Passive transport by Aquaporins(R) | 0,0007 | 8 | 2 | 0,0105 | 0,0261 | AQP4,AQP1 | |
| Integrin-linked kinase signaling(N) | 0,0041 | 45 | 4 | 0,0113 | 0,0261 | JUN,ZEB1,GIT2,ILK | |
| agrin in postsynaptic differentiation(B) | 0,0041 | 45 | 4 | 0,0113 | 0,0261 | JUN,JAK2,NRG1,NRG3 | |
| Hematopoietic cell lineage(K) | 0,0089 | 97 | 6 | 0,0113 | 0,0261 | CD14,CD24,CD36,CD34,CD44,TNF | |
| Jak-STAT signaling pathway(K) | 0,0145 | 158 | 8 | 0,0115 | 0,0261 | CNTF,LIF,JAK2,STAT1,STAT2,STAT3,RAF1,GFAP | |
| Calcineurin-regulated NFAT-dependent transcription in lymphocytes(N) | 0,0042 | 46 | 4 | 0,0121 | 0,0261 | EGR1,JUN,TNF,E2F1 | |
| Bile secretion(K) | 0,0065 | 71 | 5 | 0,0121 | 0,0261 | AQP4,AQP1,LDLR,ABCG2,ATP1A1 | |
| Inflammatory mediator regulation of TRP channels(K) | 0,0091 | 99 | 6 | 0,0124 | 0,0261 | IGF1,ASIC3,TRPV1,NGF,NTRK1,TRPA1 | |
| fc epsilon receptor i signaling in mast cells(B) | 0,0023 | 25 | 3 | 0,0125 | 0,0261 | JUN,JAK2,RAF1 | |
| IL6-mediated signaling events(N) | 0,0043 | 47 | 4 | 0,013 | 0,0261 | JUN,JAK2,STAT1,STAT3 | |
| Endocrine and other factor-regulated calcium reabsorption(K) | 0,0043 | 47 | 4 | 0,013 | 0,0261 | AP2A1,AP2B1,VDR,ATP1A1 | |
| EGFR-dependent Endothelin signaling events(N) | 0,0008 | 9 | 2 | 0,0131 | 0,0261 | EGFR,EGF | |
| sodd/tnfr1 signaling pathway(B) | 0,0008 | 9 | 2 | 0,0131 | 0,0261 | TNF,TRAF2 | |
| regulation of splicing through sam68(B) | 0,0008 | 9 | 2 | 0,0131 | 0,0261 | JAK2,RAF1 | |
| Plasminogen activating cascade(P) | 0,0008 | 9 | 2 | 0,0131 | 0,0261 | MMP13,MMP3 | |
| bcr signaling pathway(B) | 0,0024 | 26 | 3 | 0,0139 | 0,0278 | JUN,JAK2,RAF1 | |
| bioactive peptide induced signaling pathway(B) | 0,0024 | 26 | 3 | 0,0139 | 0,0278 | JAK2,STAT1,STAT3 | |
| Vasopressin regulates renal water homeostasis via Aquaporins(R) | 0,0024 | 26 | 3 | 0,0139 | 0,0278 | AQP4,AQP1,AVP | |
| Notch signaling pathway(K) | 0,0044 | 48 | 4 | 0,0139 | 0,0279 | ADAM17,NOTCH3,HDAC2,HES1 | |
| HIF-1 signaling pathway(K) | 0,0095 | 103 | 6 | 0,0147 | 0,0295 | PDK1,IGF1,TLR4,EGFR,STAT3,EGF | |
| Cocaine addiction(K) | 0,0045 | 49 | 4 | 0,0149 | 0,0298 | JUN,TH,BDNF,CDK5 | |
| Neurotransmitter Receptor Binding And Downstream Transmission In The Postsynaptic Cell(R) | 0,0123 | 134 | 7 | 0,0151 | 0,0301 | BRAF,AP2A1,NEFL,AP2B1,NSF,RAF1,MDM2 | |
| cAMP signaling pathway(K) | 0,0184 | 200 | 9 | 0,0153 | 0,0306 | SOX9,JUN,BRAF,NPY,RAF1,BDNF,AMH,PDE4D,ATP1A1 | |
| Amine-derived hormones(R) | 0,0009 | 10 | 2 | 0,0159 | 0,0319 | TH,DBH | |
| p53 pathway by glucose deprivation(P) | 0,0009 | 10 | 2 | 0,0159 | 0,0319 | TP63,TP53 | |
| Viral carcinogenesis(K) | 0,0187 | 203 | 9 | 0,0167 | 0,0333 | TP53,JUN,NRAS,STAT3,MDM2,TBP,TRAF2,HDAC2,BAX | |
| Dorso-ventral axis formation(K) | 0,0026 | 28 | 3 | 0,0169 | 0,0338 | BRAF,EGFR,NOTCH3 | |
| Notch signaling pathway(N) | 0,0048 | 52 | 4 | 0,0181 | 0,0362 | YY1,ADAM10,ADAM12,NOTCH3 | |
| Signaling by MET(R) | 0,0048 | 52 | 4 | 0,0181 | 0,0362 | NRAS,STAT3,HGF,CBL | |
| Insulin signaling pathway(K) | 0,0129 | 140 | 7 | 0,0186 | 0,0373 | BRAF,FASN,NRAS,IRS2,TSC2,RAF1,CBL | |
| ALK2 signaling events(N) | 0,001 | 11 | 2 | 0,019 | 0,0381 | BMP7,AMH | |
| Signaling by NOTCH3(R) | 0,001 | 11 | 2 | 0,019 | 0,0381 | ADAM10,NOTCH3 | |
| TGF-beta signaling pathway(P) | 0,0074 | 80 | 5 | 0,0193 | 0,0386 | JUN,BMP7,BMP4,BMP2,GDNF | |
| Signaling events mediated by Hepatocyte Growth Factor Receptor (c-Met)(N) | 0,0074 | 80 | 5 | 0,0193 | 0,0386 | EGR1,JUN,HGF,RAF1,CBL | |
| hiv-1 nef: negative effector of fas and tnf(B) | 0,0028 | 30 | 3 | 0,0202 | 0,0404 | FADD,TNF,TRAF2 | |
| IGF1 pathway(N) | 0,0028 | 30 | 3 | 0,0202 | 0,0404 | IGF1,IRS2,RAF1 | |
| Basal cell carcinoma(K) | 0,0051 | 55 | 4 | 0,0217 | 0,0434 | TP53,SHH,BMP4,BMP2 | |
| Legionellosis(K) | 0,0051 | 55 | 4 | 0,0217 | 0,0434 | CD14,TLR4,TLR2,TNF | |
| Aurora A signaling(N) | 0,0029 | 31 | 3 | 0,022 | 0,044 | TP53,GIT1,MDM2 | |
| IL12 signaling mediated by STAT4(N) | 0,0029 | 31 | 3 | 0,022 | 0,044 | JUN,STAT3,ETV5 | |
| Serotonergic synapse(K) | 0,0104 | 113 | 6 | 0,0221 | 0,0441 | APP,BRAF,NRAS,RAF1,GNB4,TRPC1 | |
| il-10 anti-inflammatory signaling pathway(B) | 0,0011 | 12 | 2 | 0,0224 | 0,0448 | JAK2,TNF | |
| erythropoietin mediated neuroprotection through nf-kb(B) | 0,0011 | 12 | 2 | 0,0224 | 0,0448 | JUN,JAK2 | |
| p53 signaling pathway(B) | 0,0011 | 12 | 2 | 0,0224 | 0,0448 | PCNA,MDM2 | |
| Signaling events mediated by HDAC Class I(N) | 0,0052 | 56 | 4 | 0,023 | 0,046 | STAT3,TNF,YY1,HDAC2 | |
| Syndecan-2-mediated signaling events(N) | 0,0029 | 32 | 3 | 0,0239 | 0,0477 | NF1,MMP2,BAX | |
| Acute myeloid leukemia(K) | 0,0052 | 57 | 4 | 0,0243 | 0,0487 | BRAF,NRAS,STAT3,RAF1 | |
